# Supplementary material for: Epithelial-Cell-Derived Phospholipase A2 Group 1B Is an Endogenous Anthelmintic
Source: Cell Host Microbe. 2017 Oct 11;22(4):484–493.e5. doi: 10.1016/j.chom.2017.09.006 (PMC5644720; doi:10.1016/j.chom.2017.09.006)
Supplement: Document S1. Figures S1–S6 and Tables S1–S4 [file mmc1.pdf]

Cell Host & Microbe, Volume *22*

Supplemental Information

Epithelial-Cell-Derived Phospholipase A_2 Group 1B Is an Endogenous Anthelmintic

Lewis J. Entwistle, Victoria S. Pelly, Stephanie M. Coomes, Yashaswini Kannan, Jimena Perez-Lloret, Stephanie Czieso, Mariana Silva dos Santos, James I. MacRae, Lucy Collinson, Abdul Sesay, Nikolay Nikolov, Amina Metidji, Helena Helmby, David Y. Hui, and Mark S. Wilson

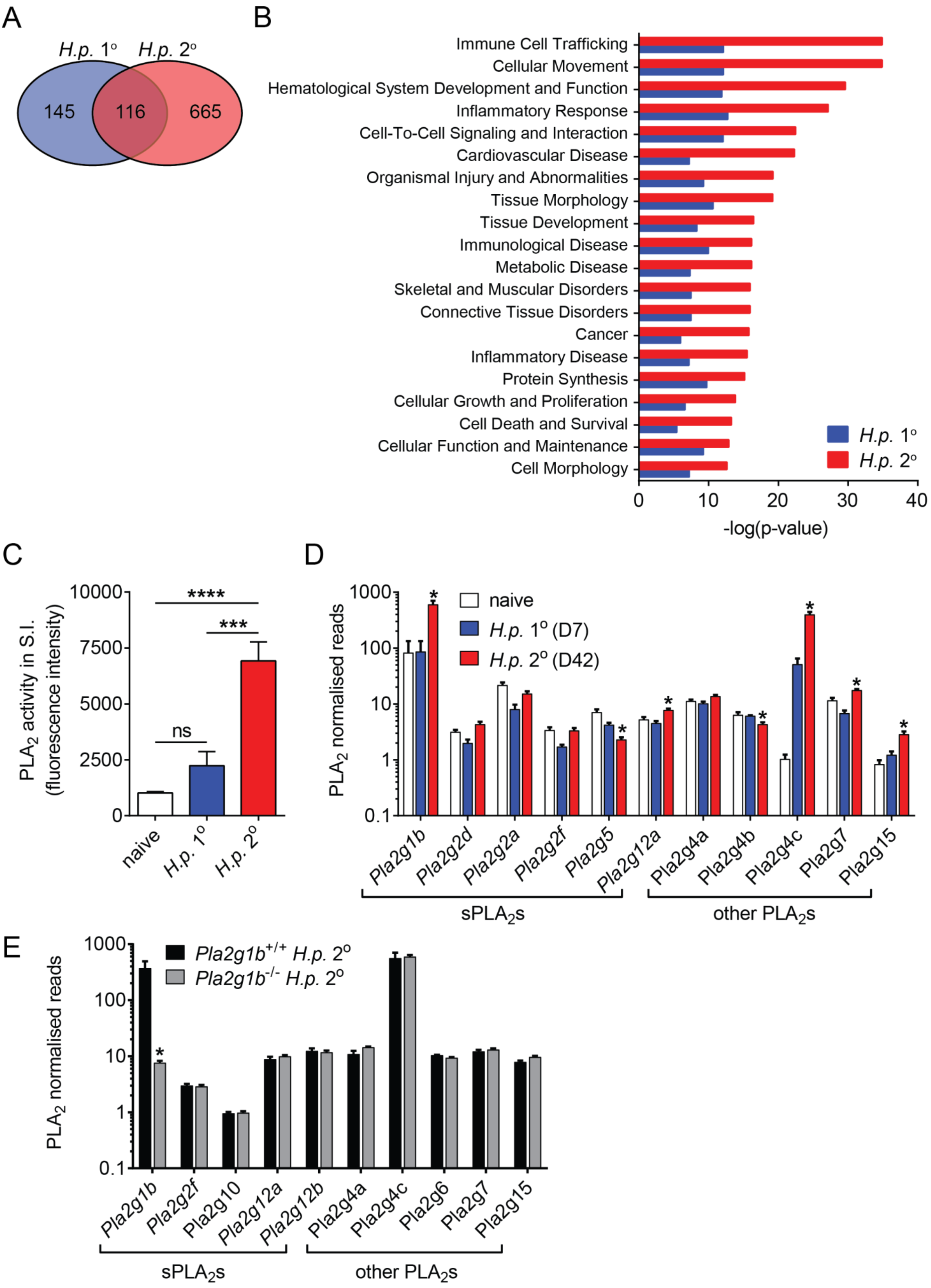
A
H.p. 1°
H.p. 2°
145
116
665
B
Immune Cell Trafficking
Cellular Movement
Hematological System Development and Function
Inflammatory Response
Cell-To-Cell Signaling and Interaction
Cardiovascular Disease
Organismal Injury and Abnormalities
Tissue Morphology
Tissue Development
Immunological Disease
Metabolic Disease
Skeletal and Muscular Disorders
Connective Tissue Disorders
Cancer
Inflammatory Disease
Protein Synthesis
Cellular Growth and Proliferation
Cell Death and Survival
Cellular Function and Maintenance
Cell Morphology
H.p. 1°
H.p. 2°
0 10 20 30 40
-log(p-value)
C
PLA2 activity in S.I. (fluorescence intensity)
10000 7500 5000 2500 0
****
***
ns
naive
H.p. 1°
H.p. 2°
D
PLA2 normalised reads
1000 100 10 1 0.1
naive
H.p. 1° (D7)
H.p. 2° (D42)
Pla2g1b Pla2g2d Pla2g2a Pla2g2f Pla2g5 Pla2g12a Pla2g4a Pla2g4b Pla2g4c Pla2g7 Pla2g15
sPLA2s
other PLA2s
E
PLA2 normalised reads
1000 100 10 1 0.1
Pla2g1b+/+ H.p. 2°
Pla2g1b-/- H.p. 2°
Pla2g1b Pla2g2f Pla2g10 Pla2g12a Pla2g12b Pla2g4a Pla2g4c Pla2g6 Pla2g7 Pla2g15
sPLA2s
other PLA2s

Figure S1. RNA sequencing analysis of 1º and 2º *H. polygyrus* infection. Related to Figure 1. (A) Common and differentially expressed genes between *H.p.* 1º and *H.p.* 2º (relative to naïve, 2-fold filter, $p<0.05$). (B) Top 20 pathways predicted to be activated in *H.p.* 2º (relative to strain naïve, 2-fold filter, $p<0.05$). (**C**) PLA_2 activity in the small intestine of naïve, *H.p.* 1º and *H.p.* 2º mice. (D & E) Phospholipase A_2 isoform expression in the small intestine from RNA sequencing. Data represented as mean ± SEM, n=6. ns = not significant, *** = $p<0.001$, **** = $p<0.0001$ determined using a one-way ANOVA with Tukey's multiple comparison analysis.

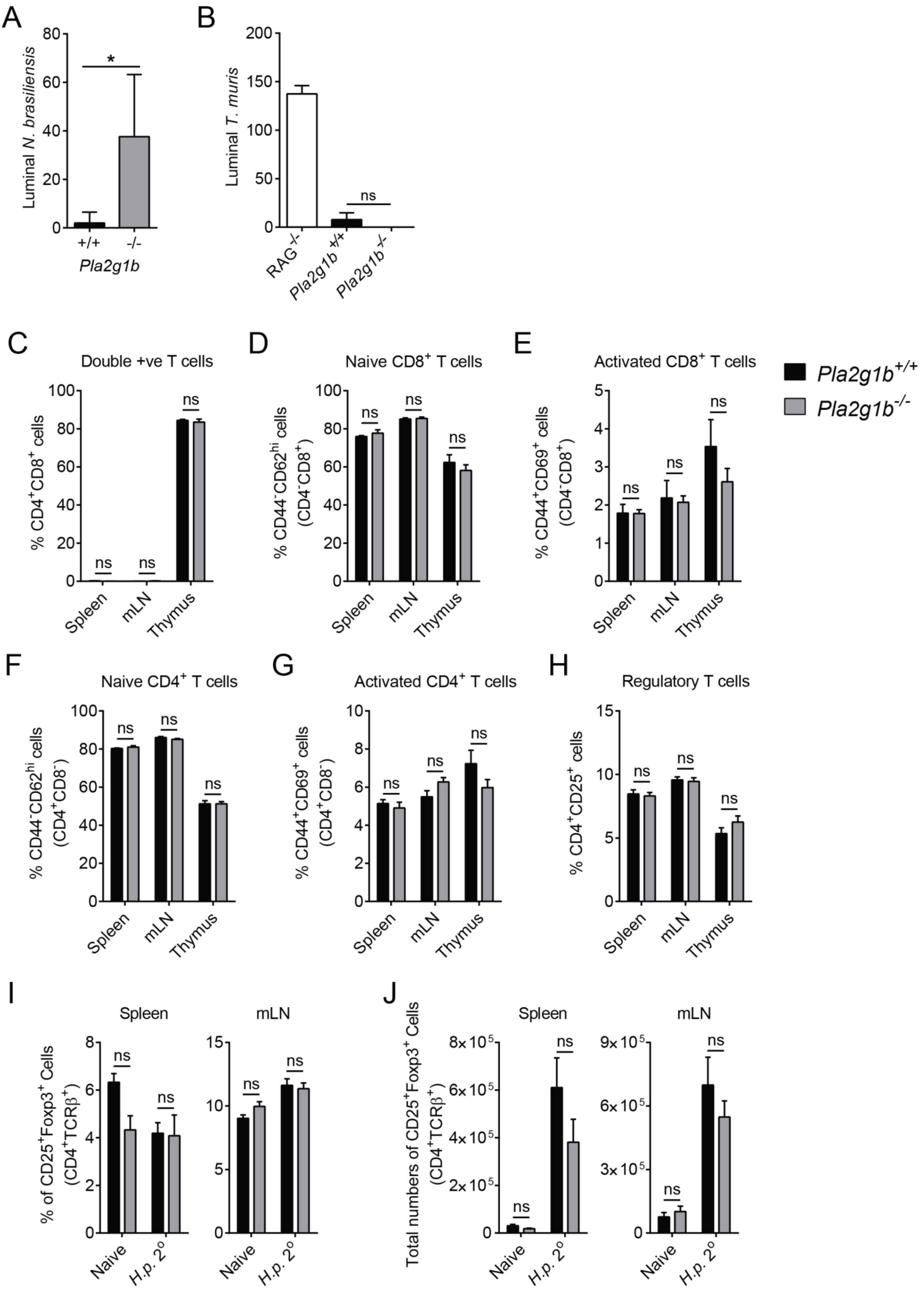

A
Luminal N. brasiliensis
+/+
-/-
Pla2g1b
B
Luminal T. muris
RAG-/-
Pla2g1b+/+
Pla2g1b-/-
ns
C
Double +ve T cells
% CD4+CD8+ cells
D
Naive CD8+ T cells
% CD44-CD62hi cells (CD4-CD8+)
E
Activated CD8+ T cells
% CD44+CD69+ cells (CD4-CD8+)
Pla2g1b+/+
Pla2g1b-/-
F
Naive CD4+ T cells
% CD44-CD62hi cells (CD4+CD8-)
G
Activated CD4+ T cells
% CD44+CD69+ cells (CD4+CD8-)
H
Regulatory T cells
% CD4+CD25+ cells
Spleen
mLN
Thymus
I
Spleen
mLN
% of CD25+Foxp3+ Cells (CD4+TCRβ+)
Naive
H.p. 2o
J
Total numbers of CD25+Foxp3+ Cells (CD4+TCRβ+)

Figure S2. *Pla2g1b* is required for effective immunity to small intestine-dwelling helminths and T cells are intact in *Pla2g1b*$^{-/-}$ mice. Related to Figure 2.

(A) Luminal *N. brasiliensis* worms in the small intestine 8 days-post infection, n=5. (B) Luminal *T. muris* worms in the cecum and large intestine 35 days-post infection. *Rag*$^{-/-}$ mice were used as an additional control to confirm the infectious dose administered. T cell frequency was assessed in naïve mice (C-J). (C) $CD4^{+}CD8^{+}$ cells. (D) $CD44^{-}CD62^{hi}$ $CD8^{+}$ cells. (E) $CD44^{+}CD69^{+}$ $CD8^{+}$ cells. (F) $CD44^{-}CD62^{hi}$ $CD4^{+}$ cells. (G) $CD44^{+}CD69^{+}$ $CD4^{+}$ cells. (H) $CD4^{+}CD25^{+}$ cells. (I) Frequency and total number of $CD25^{+}Foxp3^{+}$ $CD4^{+}TCR\beta^{+}$ cells in the spleen and (J) mLN in naïve and 7 days-post 2° *H.* polygyrus infected mice. Data represented as mean ± SEM, n=5-6. All data is representative of at least two independent experiments. ns = not significant, * = p<0.05, determined using an unpaired two-tailed t test.

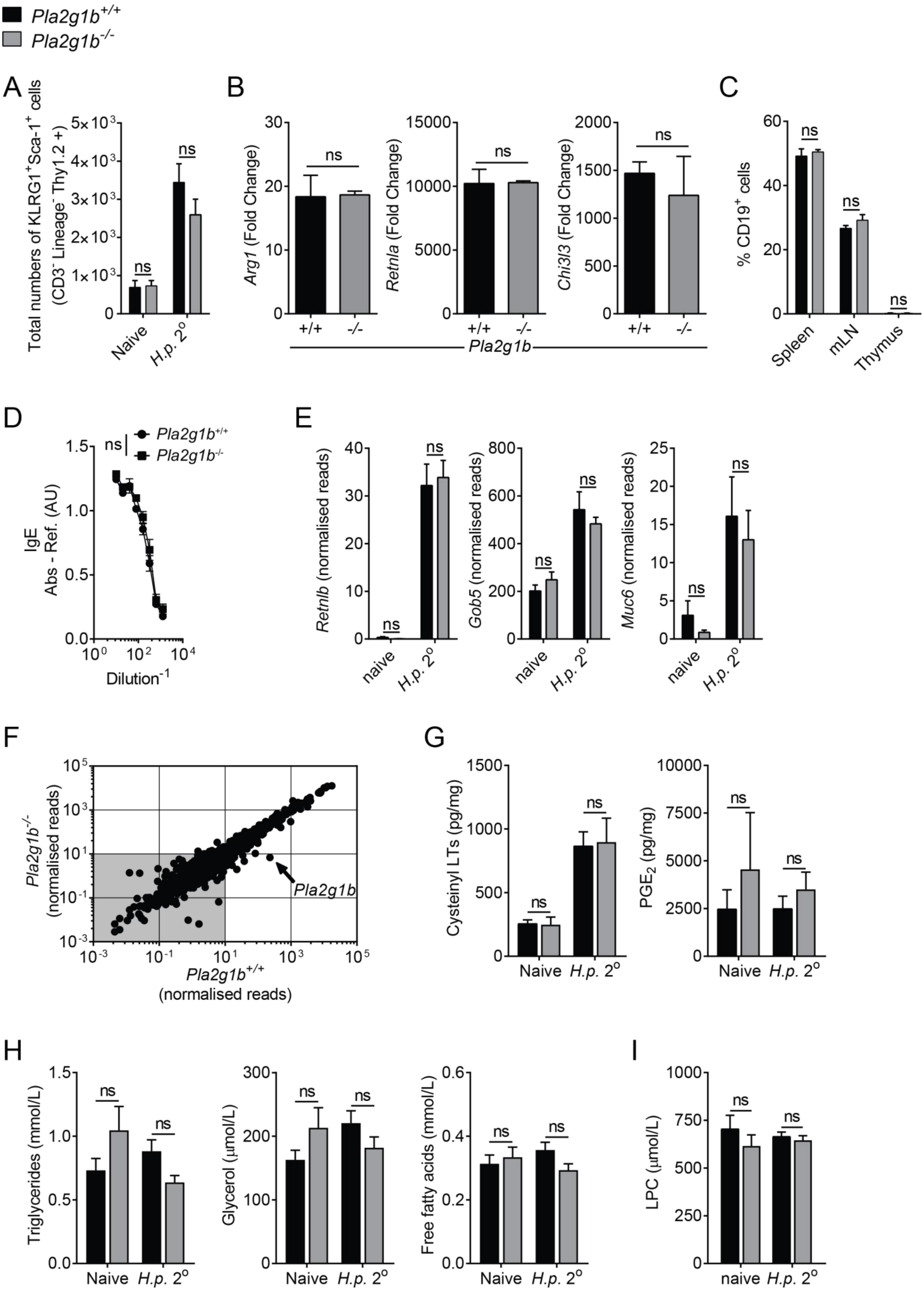

Pla2g1b+/+
Pla2g1b-/-
A
Total numbers of KLRG1+Sca-1+ cells (CD3- Lineage- Thy1.2 +)
ns
Naive
H.p. 2o
B
Arg1 (Fold Change)
Retnla (Fold Change)
Chi3l3 (Fold Change)
+/+
-/-
Pla2g1b
C
% CD19+ cells
Spleen
mLN
Thymus
D
IgE Abs - Ref. (AU)
Dilution-1
E
Retnlb (normalised reads)
Gob5 (normalised reads)
Muc6 (normalised reads)
naive
F
Pla2g1b-/- (normalised reads)
Pla2g1b+/+ (normalised reads)
Pla2g1b
G
Cysteinyl LTs (pg/mg)
PGE2 (pg/mg)
H
Triglycerides (mmol/L)
Glycerol (µmol/L)
Free fatty acids (mmol/L)
I
LPC (µmol/L)

Figure S3. Type 2 immunity, Bioactive lipids synthesis and lipid metabolism is intact in *Pla2g1b*$^{-/-}$ mice. Related to Figure 2.

(A) Total numbers of KLRG1^{+}Sca-1^{+}Lin^{-} cells in the mLN of naïve and 7 days-post 2º *H.* polygyrus infected mice. (B) Bone marrow-derived macrophages were stimulated with rIL-4 and rIL-13 for 24 hours and *Arg1*, *Retnla* and *Chi3l3* gene expression analysed. (**C**) Frequency of CD19^{+} cells were assessed in naïve mice. (D) IgE in the serum from mice 7 days-post 2º *H.* polygyrus infection. (E) Mucus-associated gene expression in the small intestine of WT and *Pla2g1b*$^{-/-}$ naïve and 7 days-post 2º *H.* polygyrus infected mice. (F) RNA sequencing-generated transcriptional landscape of the small intestine of naïve WT and *Pla2g1b*$^{-/-}$ mice (G) Cysteinyl leukotrienes (LTs) and Prostaglandin E_2 (PGE_2) concentration in the small intestine of naïve and 7 days-post 2º *H.* polygyrus infected mice. (H) Lipid metabolites in the serum of naïve and 7 days-post 2º *H.* polygyrus infected mice. (I) Lysophosphatidylcholine concentration in the serum of naïve and 7 days-post 2º *H.* polygyrus infected mice. Data represented as mean ± SEM, n=4-6. All data is representative of at least two independent experiments. ns = not significant, determined using a two-way ANOVA with Sidak's multiple comparison analysis or an unpaired two-tailed t test.

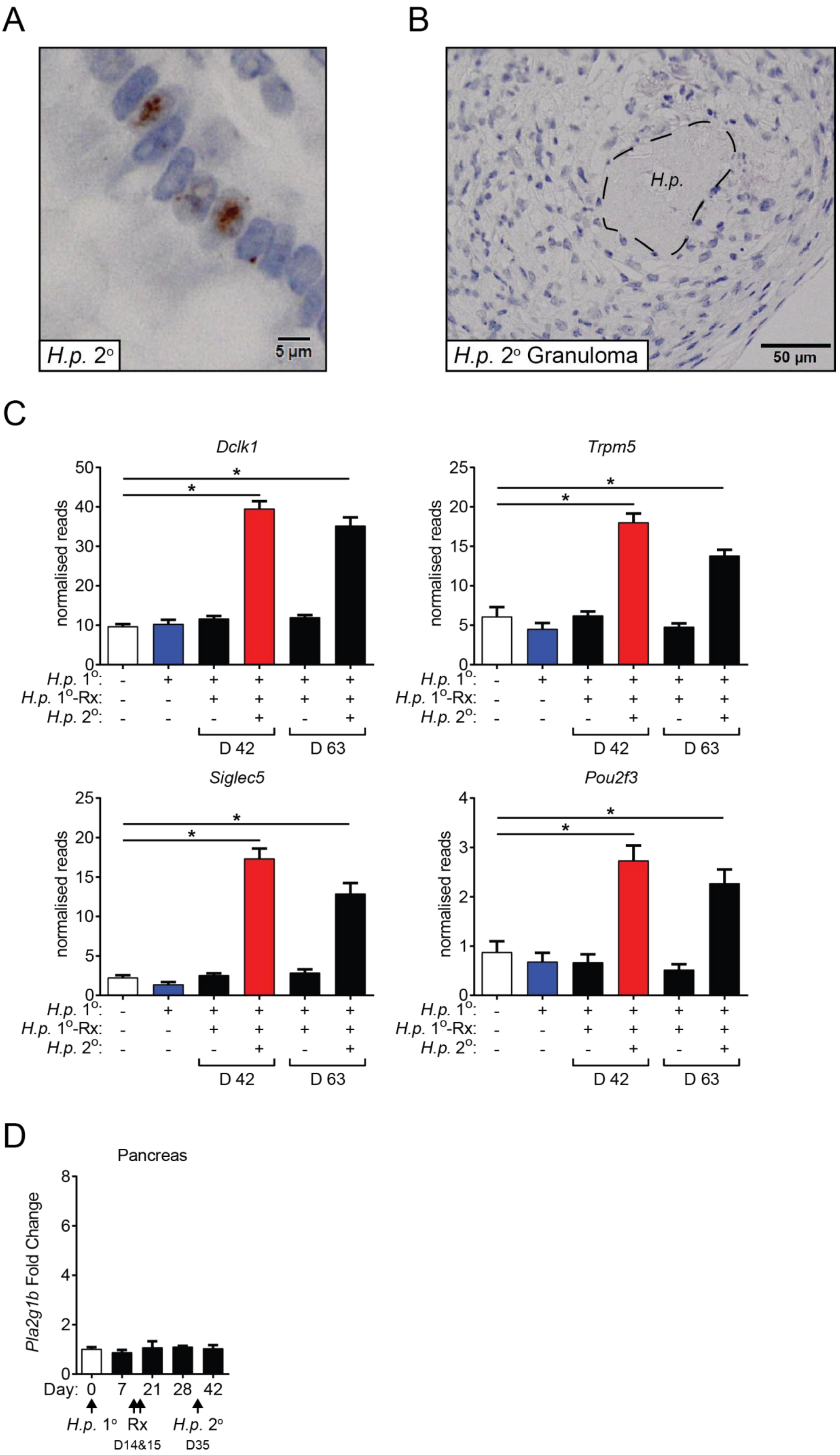
A
H.p. 2°
5 µm
B
H.p.
H.p. 2° Granuloma
50 µm
C
Dclk1
Trpm5
Siglec5
Pou2f3
normalised reads
H.p. 1°:
H.p. 1°-Rx:
H.p. 2°:
D 42
D 63
D
Pancreas
Pla2g1b Fold Change
Day: 0 7 21 28 42
H.p. 1°
Rx
D14&15
H.p. 2°
D35

Figure S4. *Pla2g1b* expression is restricted to epithelial cells in the small intestine of resistant mice. Related to Figure 4.

(A) *Pla2g1b* detection by RNAScope® ISH in the small intestine 7 days-post 2º *H.* polygyrus infection. (B) *Pla2g1b* detection by RNAScope® ISH in the granuloma surrounding *H. polygyrus* (*H.p.*) within the small intestine 7 days-post 2º *H.* polygyrus infection. (C) Tuft cell-specific gene expression data in small intestine from RNA sequencing data. (D) Kinetics of *Pla2g1b* expression in the pancreas during *H. polygyrus* 2º infection model, no significant difference detected using one-way ANOVA. Data represented as mean ± SEM, n=6-8. All data is representative of at least two independent experiments. * = $p<0.05$ determined within RNAseq analysis.

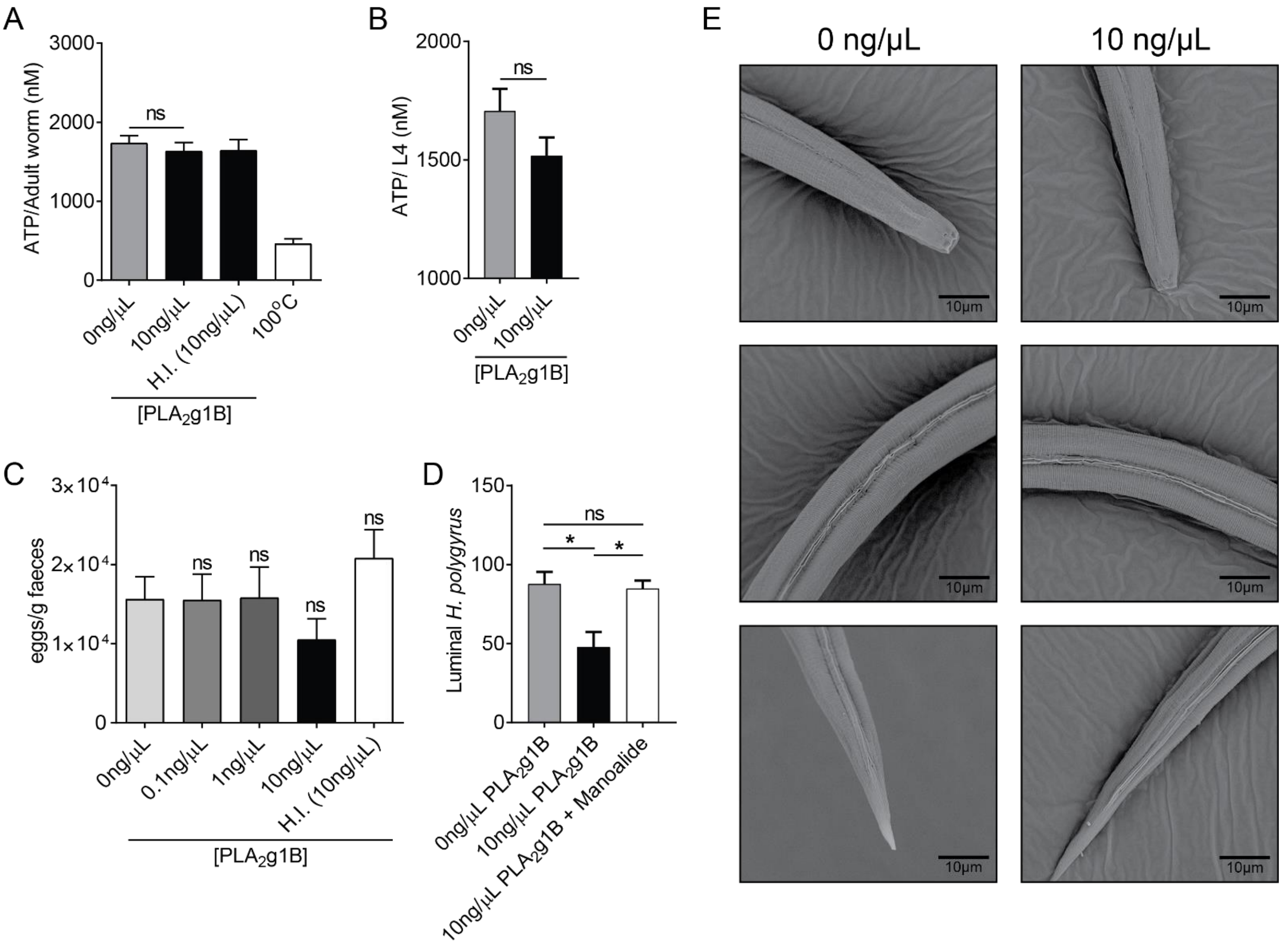

Figure S5. PLA$_2$g1B has direct anthelmintic properties against *H. polygyrus* L3 larvae. Related to Figure 3.

(A) ATP concentration of adult *H. polygyrus* worms following 24-hour treatment with rPLA$_2$g1B *in vitro*. (B) ATP concentration of L4 *H. polygyrus* isolated from the small intestine 14 days-post 1º infection following 24-hour treatment with rPLA$_2$g1B, n=40 (data pooled from three independent experiments). (C) Eggs recovered in the faeces 14 days-post 1º infection following 24-hour treatment with rPLA$_2$g1B, n=10 (data pooled from two independent experiments). (D) Luminal *H. polygyrus* worms in the small intestine 14 days-post 1º infection following 24-hour treatment with rPLA$_2$g1B ± Manoalide (200 ng ng/µL). (E) SEM of L3 *H.* polygyrus larvae after treatment with rPLA$_2$g1B. Data represented as mean ± SEM, n=5. All data is representative of at least two independent experiments. ns = not significant, * = p<0.05 determined using a one-way ANOVA with Tukey's or Dunnett's multiple comparison analysis or an unpaired two-tailed t-test.

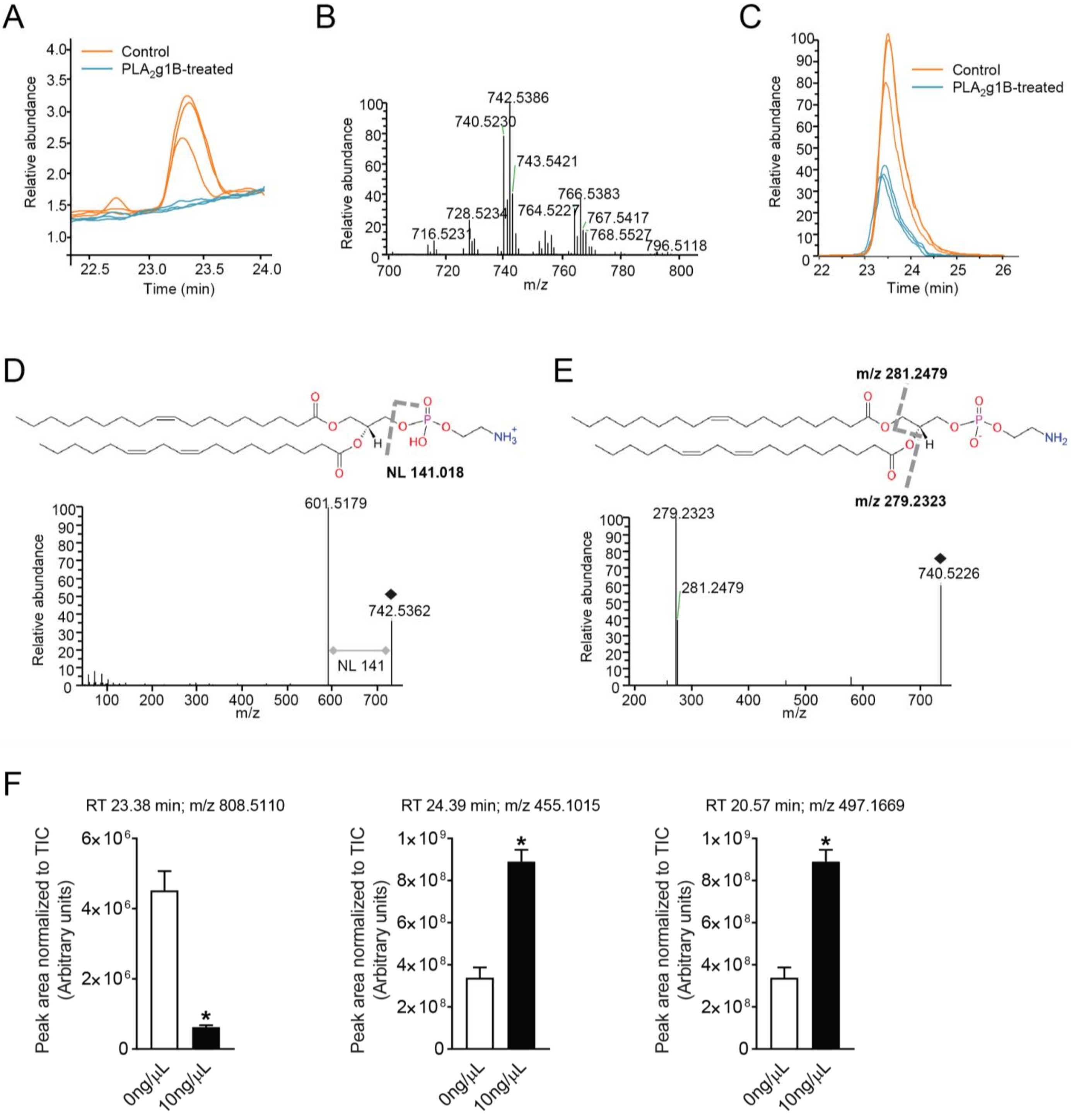

A
Control
PLA2g1B-treated
Relative abundance
Time (min)
B
742.5386
740.5230
743.5421
766.5383
764.5227
767.5417
728.5234
716.5231
768.5527
796.5118
Relative abundance
m/z
C
Control
PLA2g1B-treated
Relative abundance
Time (min)
D
NL 141.018
601.5179
742.5362
NL 141
Relative abundance
m/z
E
m/z 281.2479
m/z 279.2323
279.2323
281.2479
740.5226
Relative abundance
m/z
F
RT 23.38 min; m/z 808.5110
RT 24.39 min; m/z 455.1015
RT 20.57 min; m/z 497.1669
Peak area normalized to TIC (Arbitrary units)
0ng/µL
10ng/µL

Figure S6. Identification of phosphatidylethanolamine (PE) species by LC-MS/MS. Related to Figure 6.

(A) Relevant section of the base peak chromatogram of control and Pla2g1b-treated larvae showing elution of PE phospholipids in negative ion mode, n=3. (B) Example spectrum form the section of the chromatogram in (A) at the approximate retention time of PE species 23.23-23.40 minutes. PEs consist of a phosphoethanolamine head group attached to a glycerol backbone, itself attached to two fatty acid moieties via phosphoether and ester bonds, respectively. As a result, PE MS spectra display traits of fatty acid-containing molecules (clusters that have inter-cluster mass shifts of 28 Da (CH_2CH_2) and intra-cluster mass shifts of 2 Da (indicative of difference in double bond number (fatty acid saturation)). The six identified and three putatively identified PEs are shown in Figure 6A, B. (C) Extracted ion chromatogram of the ion corresponding to PE 36:3 (18:1, 18:2) in negative ion mode (m/*z* 740.5226). (D) Positive-ion MS/MS was used to confirm assignment of the peak as PE, where neutral loss of 141 Da indicates phopshoethanolamine head group. Here, we show the fragmentation of the ion corresponding to PE 36:3 (18:1, 18:2) in positive ion mode (m/*z* 742.5362). (E) Negative ion fragmentation of the ion corresponding to PE 36:3 (18:1, 18:2) (m/*z* 740.5226). By identifying both fatty moieties, the individual molecular species were recognized (in this instance, 18:1 and 18:2). Both, the arrangement of the fatty acid moieties at the glycerol backbone (i.e. *sn*-1 or *sn*-2) and the position of the double bonds could not be inferred. (F) Relative abundances of unidentified lipid features extracted from PLA_2g1B-treated (10 ng/µL) and control-treated (0 ng/µL), n=3. Data is shown as normalised intensities expressed in arbitrary units. Data represented as mean ± SEM. TIC: Total ion current.

Cluster 1: Genes	*H.p.* 2º vs *H.p.* 1º Fold change	*H.p.* 1º Fold change (rel. to naïve)	*H.p.* 2º Fold change (rel. to naïve)
Chi3l3/Chi3l4	31.57018	126.558	3995.459
GSDMC	17.25631	67.532	1165.353
SPP1	12.70641	5.024	63.837
RNASE2	12.13344	373.801	4535.492
Retnlb	7.871389	66.013	519.614
Ccl8	7.796338	11.632	90.687
PLA2G4C	7.76945	49.434	384.075
Retnla	7.459337	73.334	547.023
ARG1	5.484869	24.023	131.763
TIMP1	5.245189	11.432	59.963
OLR1	5.064237	5.542	28.066
CCL24	4.347589	14.474	62.927
Irg1	3.618186	17.986	4.971
CCL7	3.5964	8.444	30.368
F7	3.310224	25.127	83.176
CPXM1	3.203643	5.161	16.534
Ccl2	2.565859	8.465	21.72
HP	2.459766	12.502	30.752
Ear2 (includes others)	2.434257	5.126	12.478
F13A1	2.262129	5.524	12.496
TREM2	2.22541	8.225	18.304
Cbr2	2.195861	10.533	23.129
SERPINA3	2.081023	8.368	17.414
C3	2.007541	5.172	10.383
Saa3	1.967481	9.779	19.24
2310042E22Rik (includes others)	1.731672	11.758	20.361
Scd2	1.318527	5.729	4.345
SELL	1.05153	8.694	9.142

Table S1. Cluster 1 gene list. Related to Figure 1.

Genes in cluster 1 as determined in Fig. 1c (Filtered on >5 fold in *H.p.* 1º (relative to naïve), $p<0.05$).

Cluster 2: Genes	*H.p.* 2º vs *H.p.* 1º Fold change	*H.p.* 1º Fold change (rel. to naïve)	*H.p.* 2º Fold change (rel. to naïve)
1810009J06Rik/Gm2663	162.088	1	162.088
Mcpt1	74.305	1	74.305
Mcpt2	59.661	1	59.661
REG1B	45.654	1	45.654
Reg3d	33.858	1	33.858
CPA3	32.831	1	32.831
SPTA1	28.655	1	28.655
mir-675	26.276	1	26.276
FETUB	22.682	1	22.682
Rab44	19.101	1	19.101
PPY	17.489	1	17.489
SLC7A2	17.028	1	17.028
RANBP3L	16.868	1	16.868
PAH	16.531	1	16.531
CUZD1	16.173	1	16.173
ITIH4	15.567	1	15.567
PRSS3	14.71	1	14.71
SPRR2G	12.941	1	12.941
AMY2A	12.738	1	12.738
Prss2	12.642	1	12.642
PAQR9	12.445	1	12.445
Try4/Try5	12.386	1	12.386
DCDC2	12.242	1	12.242
GC	11.945	1	11.945
Tmed11	11.423	1	11.423
GGH	11.2	1	11.2
SYCN	11.142	1	11.142
CEL	11.14	1	11.14
SERPINI2	10.909	1	10.909
AMY1A (includes others)	10.779	1	10.779
CCL2	10.591	1	10.591
KLK3	10.557	1	10.557
GP2	10.119	1	10.119
CELA3A	9.918	1	9.918
PTF1A	9.807	1	9.807
SOSTDC1	9.777	1	9.777
CTRB2	9.712	1	9.712
CPA1	9.672	1	9.672
TMED6	9.625	1	9.625
CCKAR	9.609	1	9.609
Gm5409/Try10	9.585	1	9.585
CLDN10	9.084	1	9.084
AASS	8.706	1	8.706
RNASE1	8.567	1	8.567
PTGER3	8.199	1	8.199
SLC38A3	8.189	1	8.189
PNLIPRP2	7.977	1	7.977
CBS	7.932	1	7.932

RETNLB	7.896	1	7.896
Amy2b	7.83	1	7.83
ALOX15	7.823	1	7.823
CLPS	7.77	1	7.77
LOC100862462	7.684	1	7.684
Siglec5	7.652	1	7.652
GATM	7.619	1	7.619
ERP27	7.596	1	7.596
ANGPT1	7.54	1	7.54
SLC34A2	7.502	1	7.502
TNIP3	7.492	1	7.492
MUC6	7.477	1	7.477
CPB1	7.448	1	7.448
C8orf47	7.352	1	7.352
CASP9	7.349	1	7.349
SH2D6	7.318	1	7.318
PLA2G1B	7.292	1	7.292
LRRC7	7.23	1	7.23
HAPLN4	7.132	1	7.132
Igk	7.123	1	7.123
CHST2	7.106	1	7.106
NUPR1	6.785	1	6.785
RGS22	6.752	1	6.752
VTN	6.715	1	6.715
PNLIPRP1	6.651	1	6.651
AQP12A/AQP12B	6.598	1	6.598
GABRA4	6.592	1	6.592
IAPP	6.59	1	6.59
LRRN1	6.483	1	6.483
Ighg2c	6.409	1	6.409
ARHGDIG	6.349	1	6.349
Hamp/Hamp2	6.31	1	6.31
IGF1	6.273	1	6.273
TMEM108	6.273	1	6.273
TFF2	6.261	1	6.261
Wfdc18	6.256	1	6.256
Ang2 (includes others)	6.214	1	6.214
TREML2	6.161	1	6.161
LY6G6F	6.114	1	6.114
Clu	6.113	1	6.113
CTSV	6.068	1	6.068
SH2D7	6.062	1	6.062
CELA3B	6.053	1	6.053
CILP	5.948	1	5.948
ALOX5AP	5.947	1	5.947
NPHS1	5.924	1	5.924
GNMT	5.894	1	5.894
DHRS9	5.881	1	5.881
LCAT	5.863	1	5.863

FUT2	5.862	1	5.862
PRSS1	5.857	1	5.857
ALDH1L2	5.8	1	5.8
GPRC5C	5.729	1	5.729
FKBP11	5.711	1	5.711
MUC1	5.71	1	5.71
RNY1	5.698	1	5.698
HCK	5.663	1	5.663
TFPI2	5.645	1	5.645
G6PC	5.643	1	5.643
Igh-VS107	5.63	1	5.63
PDCD1LG2	5.628	1	5.628
CPA2	5.532	1	5.532
HPN	5.453	1	5.453
CLEC7A	5.45	1	5.45
GAL	5.399	1	5.399
RBPJL	5.322	1	5.322
NFIL3	5.276	1	5.276
SRGN	5.231	1	5.231
CLDN8	5.224	1	5.224
FAM221A	5.212	1	5.212
HSBP1L1	5.188	1	5.188
CELA1	5.129	1	5.129
ME1	5.12	1	5.12
CA4	5.097	1	5.097
TSPAN6	5.083	1	5.083
FN1	5.08	1	5.08
SLC38A5	5.065	1	5.065
GLIPR2	5.031	1	5.031

Table S2. Cluster 2 gene list. Related to Figure 1.

Genes in cluster 2 as determined in Fig. 1c (Filtered on >5 fold in *H.p.* 2° (relative to naïve), p<0.05).

**Cluster 3: Genes**	***H.p.* 2º vs *H.p.* 1º Fold change**	***H.p.* 1º Fold change (rel. to naïve)**	***H.p.* 2º Fold change (rel. to naïve)**
Gm11194	-33.158	1	-33.158
SLC13A2	-20.622	1	-20.622
DBP	-11.072	1	-11.072
Cyp2a12/Cyp2a22	-7.598	1	-7.598

Table S3. Cluster 3 gene list. Related to Figure 1.

Genes in cluster 3 as determined in Fig. 1c (Filtered on <-5 fold in *H.p.* 2º (relative to naïve), $p<0.05$).

Gene	Forward primer sequence	Reverse primer sequence
Hprt	GCCCTTGACTATAATGAGTACTTCAGG	TTCAACTTGCGCTCATCTTAGG
Pla2g1b	CTCGGGCCGTGTGGCAGTTC	TGCCGAGCCAGAGCACGAGT
Arg1	GGAAAGCCAATGAAGAGCTG	GCTTCCAACTGCCAGACTGT
Retnla	CCCTCCACTGTAACGAAGACTC	CACACCCAGTAGCAGTCATCC
Chi3l3	CATGAGCAAGACTTGCGTGAC	GGTCCAAACTTCCATCCTCCA
Retnlb	ATGGGTGTCACTGGATGTGCTT	AGCACTGGCAGTGGCAAGTA
Gob5	CATCGCCATAGACCACGACG	TTCCAGCTCTCGGGAATCAAA

Table S4. qPCR oligonucleotide primer sequences. Related to Experimental Procedures.
