# Supplementary material for: Epithelial-Cell-Derived Phospholipase A2 Group 1B Is an Endogenous Anthelmintic
Source: Cell Host Microbe. 2017 Oct 11;22(4):484–493.e5. doi: 10.1016/j.chom.2017.09.006 (PMC5644720; doi:10.1016/j.chom.2017.09.006)
Supplement: Document S2. Article plus Supplemental Information [file mmc2.pdf]

Cell Host & Microbe

Epithelial-Cell-Derived Phospholipase A_2 Group 1B Is an Endogenous Anthelmintic

Graphical Abstract

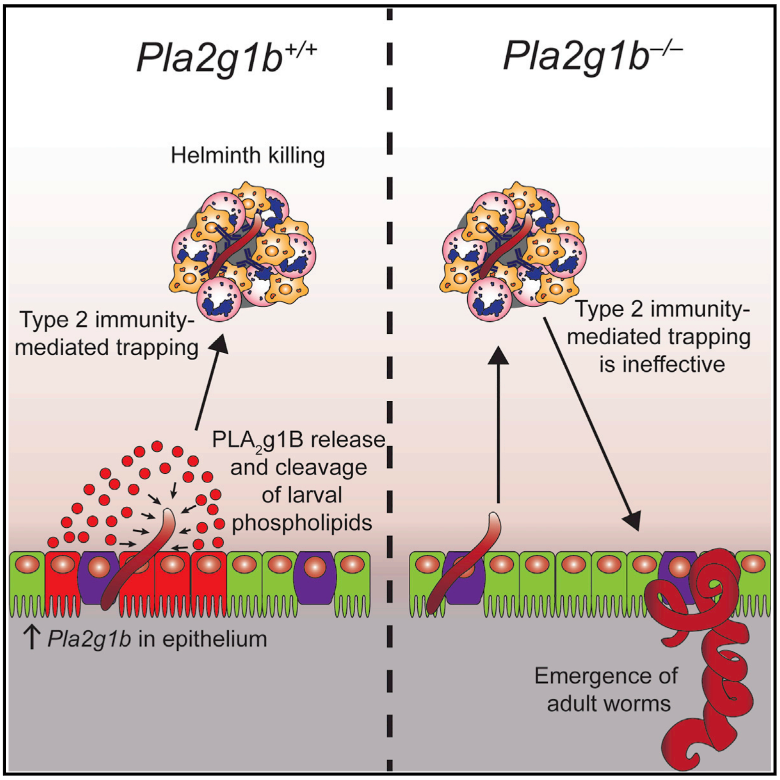

Highlights

- *Pla2g1b* expression correlated with resistance to intestinal helminth infection
- PLA_2g1B is essential for resistance to intestinal helminth infection in mice
- PLA_2g1B directly reduces phospholipid abundance in infective larvae
- *Pla2g1b* is expressed by epithelial cells and is negatively regulated by IL-4Rα

Authors

Lewis J. Entwistle, Victoria S. Pelly, Stephanie M. Coomes, ..., Helena Helmby, David Y. Hui, Mark S. Wilson

Correspondence

wilson.mark@gene.com

In Brief

Intestinal helminths are highly prevalent in developing countries, with chronic infection causing significant host morbidity. Entwistle et al. show that epithelial-derived phospholipase A_2 group 1B (PLA_2g1B) acts as an endogenous anthelmintic and is essential for resistance to intestinal helminth infection via direct action on infective larvae.

CrossMark

Entwistle et al., 2017, Cell Host & Microbe *22*, 484–493
October 11, 2017 © 2017 The Authors. Published by Elsevier Inc.
https://doi.org/10.1016/j.chom.2017.09.006

CellPress

Cell Host & Microbe
Article

Epithelial-Cell-Derived Phospholipase A_2 Group 1B Is an Endogenous Anthelmintic

Lewis J. Entwistle,[1] Victoria S. Pelly,[1] Stephanie M. Coomes,[1] Yashaswini Kannan,[1] Jimena Perez-Lloret,[1] Stephanie Czieso,[1] Mariana Silva dos Santos,[2] James I. MacRae,[2] Lucy Collinson,[3] Abdul Sesay,[4] Nikolay Nikolov,[4] Amina Metidji,[5] Helena Helmby,[6] David Y. Hui,[7] and Mark S. Wilson[1,8,9,*]
[1]Allergy and Anti-helminth Immunity Laboratory
[2]Metabolomics
[3]Electron Microscopy
[4]Advanced Sequencing Facility
[5]AhR Immunity Laboratory
The Francis Crick Institute, 1 Midland Road, London NW1 1AT, UK
[6]Department of Immunology and Infection, Faculty of Infectious and Tropical Diseases, London School of Hygiene and Tropical Medicine, Keppel Street, London, WC1E 7HT, UK
[7]Department of Pathology, Metabolic Disease Research Center, University of Cincinnati College of Medicine, Cincinnati, OH 45237, USA
[8]Immunology Discovery, Genentech Inc., South San Francisco, CA 94080, USA
[9]Lead Contact
*Correspondence: wilson.mark@gene.com
https://doi.org/10.1016/j.chom.2017.09.006

SUMMARY

Immunity to intestinal helminth infections has been well studied, but the mechanism of helminth killing prior to expulsion remains unclear. Here we identify epithelial-cell-derived phospholipase A_2 group 1B (PLA_2g1B) as a host-derived endogenous anthelmintic. PLA_2g1B is elevated in resistant mice and is responsible for killing tissue-embedded larvae. Despite comparable activities of other essential type-2-dependent immune mechanisms, *Pla2g1b*$^{-/-}$ mice failed to expel the intestinal helminths *Heligmosomoides polygyrus* or *Nippostrongylus brasiliensis*. Expression of *Pla2g1b* by epithelial cells was dependent upon intestinal microbiota, adaptive immunity, and common-gamma chain-dependent signaling. Notably, *Pla2g1b* was downregulated in susceptible mice and inhibited by IL-4R-signaling in vitro, uncoupling parasite killing from expulsion mechanisms. Resistance was restored in *Pla2g1b*$^{-/-}$ mice by treating infective *H. polygyrus* L3 larvae with PLA_2g1B, which reduced larval phospholipid abundance. These findings uncover epithelial-cell-derived *Pla2g1b* as an essential mediator of helminth killing, highlighting a previously overlooked mechanism of anti-helminth immunity.

INTRODUCTION

Intestinal helminth infections are highly prevalent in developing countries, with chronic infections causing significant host morbidity (Hotez et al., 2008). With the emergence of drug-resistant helminths, a limited number of effective anthelmintics, and stalling vaccine efforts, new therapeutic avenues require a better understanding of anti-helminth immunity and killing. Expulsion mechanisms of intestinal helminths have been widely studied and reported; however, the mechanism of helminth killing in the tissue prior to expulsion remain unclear. Upon infection, activated epithelial cells secrete a suite of alarmins, including interleukin (IL)-25, thymic stromal lymphopoietin (TSLP), and IL-33, which promote activation and differentiation of innate and adaptive immune cells, leading to type 2 inflammation (Anthony et al., 2006; Katona et al., 1991; McCoy et al., 2008; Urban et al., 1991a). IL-4-driven CD4$^+$ T helper (Th)-2 cell differentiation, in combination with group 2 innate lymphoid cell (ILC2) activation, leads to the secretion of a suite of cytokines including IL-4, IL-5, IL-9, and IL-13, which propagate type 2 inflammation and activate the local stroma (Grencis et al., 1991; Hashimoto et al., 2009). The resulting reorganization of intestinal tissue—with goblet cell hyperplasia, mucus hyper-secretion, and smooth muscle contraction (Gerbe et al., 2016; Hashimoto et al., 2009; Hasnain et al., 2010, 2011; Howitt et al., 2016; Murakami et al., 2016)—alongside type 2 cytokine-driven immunological changes such as B cell class switching (McCoy et al., 2008) and alternate activation of macrophages (Anthony et al., 2006) contributes to parasite expulsion. However, the precise mechanism of parasite damage and killing, whether in tissue or lumen, has remained unclear.

RESULTS AND DISCUSSION

To identify local tissue responses and novel mechanisms of intestinal helminth killing during anti-helminth immunity, we used the natural mouse intestinal helminth *H. polygyrus* (*H.p.*). Following oral infection, stage 3 larvae (L3) migrate to the duodenum and proximal jejunum, where they penetrate the mucosae and embed into the *muscularis externa*, undergoing developmental moults before emerging into the lumen as adult worms (Camberis et al., 2003; Valanparambil et al., 2014). C57BL/6 mice are naturally susceptible to a primary (1°) *H.p.* infection, establishing a chronic infection (Reynolds et al.,

 © 2017 The Authors. Published by Elsevier Inc.
This is an open access article under the CC BY license (http://creativecommons.org/licenses/by/4.0/).

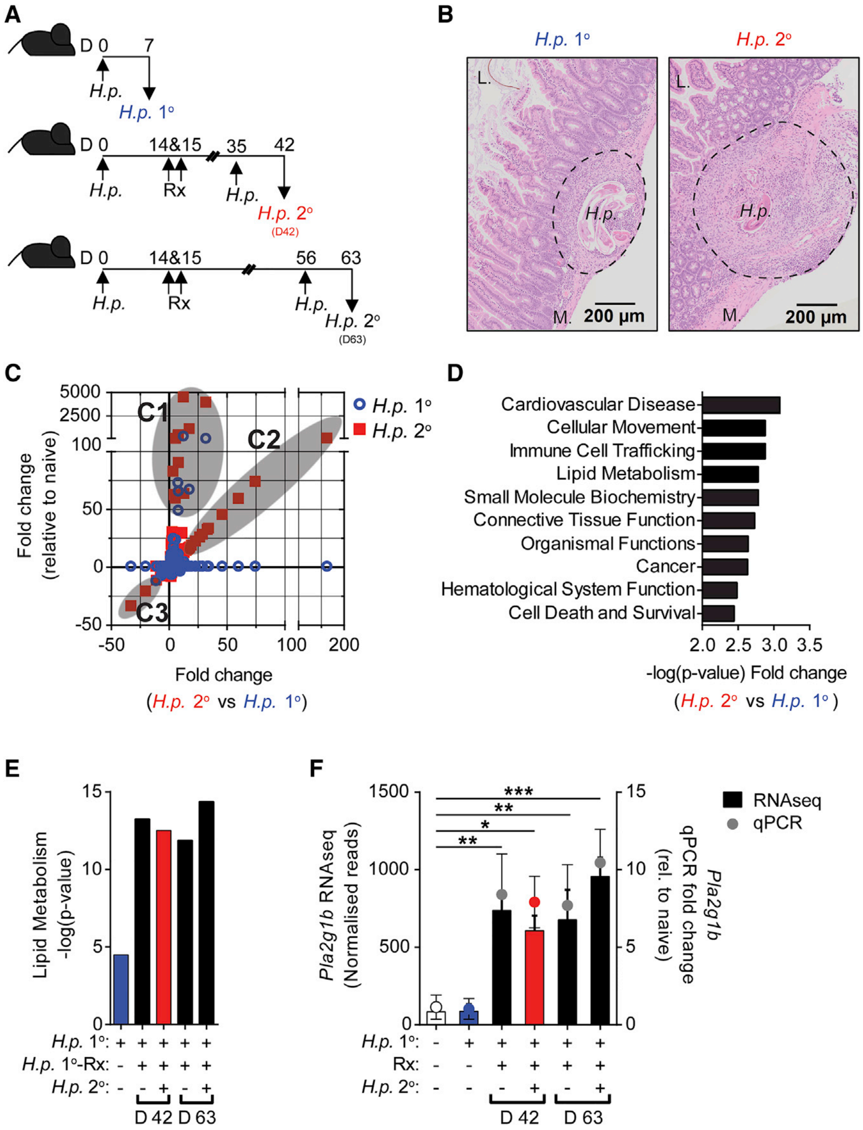

Figure 1. *Pla2g1b* and Lipid Metabolism Correlate with Resistance to Intestinal Helminth Infection

(A) C57BL/6 mice were orally infected with 200 L3 *H. polygyrus* (*H.p.*) larvae on day 0. A cohort of mice were sacrificed 7 days after 1° *H.p.* infection (*H.p.* 1°). Remaining mice were drug treated (Rx) on days 14 and 15. Mice were then reinfected with *H.p.* on day 35 or day 56 and harvested 7 days after infection (*H.p.* 2°).

(B) H&E staining of the small intestine from *H.p.* 1° and *H.p.* 2° (D42).

(C) Ratio-of-ratios analysis of differentially expressed genes in *H.p.* 1° and *H.p.* 2° (D42) identified distinct gene clusters (C1-3).

(D) Top 10 pathways predicted to be activated more highly in and *H.p.* 2° than *H.p.* 1° (both relative to naive, 2-fold filter, $p < 0.05$).

(E) Lipid metabolism pathway predicted activation score (relative to naive, 2-fold filter, $p < 0.05$).

(F) *Pla2g1b* expression in small intestine from RNA sequencing data, confirmed by qPCR.

Data are represented as mean ± SEM; n = 8, * = $p < 0.05$, ** = $p < 0.01$ determined using a one-way ANOVA with Dunnett's multiple comparison analysis. See also Figure S1 and Tables S1–S3.

2012). Following drug cure of a 1° infection (Rx), C57BL/6 mice are resistant to a secondary (2°) *H.p.* challenge infection (Finkelman et al., 1997; model, Figure 1A). Resistance to *H.p.* 2° infection correlated with substantial inflammation and tissue remodeling (Figure 1B), and significantly more transcriptional activity in duodenal tissue than in 1° infection (*H.p.* 1°), with 665 genes differentially expressed in 2° infection compared to 145 genes in 1° infection and 116 common genes (relative to naive, 2-fold filter, $p < 0.05$) (Figure S1A). Using a ratio-of-ratios analysis to specifically identify genes expressed in resistant mice (*H.p.* 2°), we identified three transcriptional clusters based on their expression relative to uninfected mice and relative to susceptible mice (*H.p.* 1°) (Figure 1C, C1-C3; Tables S1–S3). Cluster 1 (C1) identified common and quantitative differences between 1° and 2° infection, including several genes previously described in immunity to *H.p.* (Anthony et al., 2006; Herbert et al., 2009). Cluster 2 (C2) identified qualitative differences between susceptible and resistant mice highlighting genes upregulated in *H.p.* 2° infection only. Many of these genes have not previously been described in immunity to *H.p.* Cluster 3 (C3) identified qualitative differences downregulated in *H.p.* 2° infection only. Pathway analysis reflected quantitative and qualitative transcriptional differences with a greater increase in immune-activated pathways in resistant mice, as previously described (Allen and Maizels, 2011; Anthony et al., 2007; Maizels et al., 2012), in addition to an increase in lipid metabolism pathways in *H.p.* 2° that was not previously described during anti-helminth immunity (Figures 1D and S1B). Increased activation of lipid metabolism pathways was also evident in resistant mice with or without a 2° challenge infection and was maintained for up to 48 days after drug treatment (Figure 1E), correlating with long-term resistance to reinfection following drug treatment (Urban et al., 1991b). Within C2 genes, which were upregulated in *H.p.* 2° infection only, we identified group 1B phospholipase A_2, *Pla2g1b*, a member of a large family of secreted ($sPLA_2$) enzymes that regulate lipid metabolism through hydrolysis of phospholipids (Labonté et al., 2006, 2010). *Pla2g1b* was significantly increased in drug-treated mice with or without challenge infection, correlating with lipid metabolism pathways and resistance to *H.p.* (Figure 1F). The enzymatic activity of PLA_2 in the small intestine was marginally increased in susceptible mice but dramatically increased in resistant mice (Figure S1C), reflecting a broad increase in several PLA_2 enzymes in resistant mice (Figure S1D).

To formally test whether elevated PLA_2g1B contributed to resistance to intestinal helminth infections, we infected *Pla2g1b*$^{-/-}$ mice with a variety of small- or large-intestinal helminths. Strikingly, *Pla2g1b* was essential for resistance to *H.p.*, with

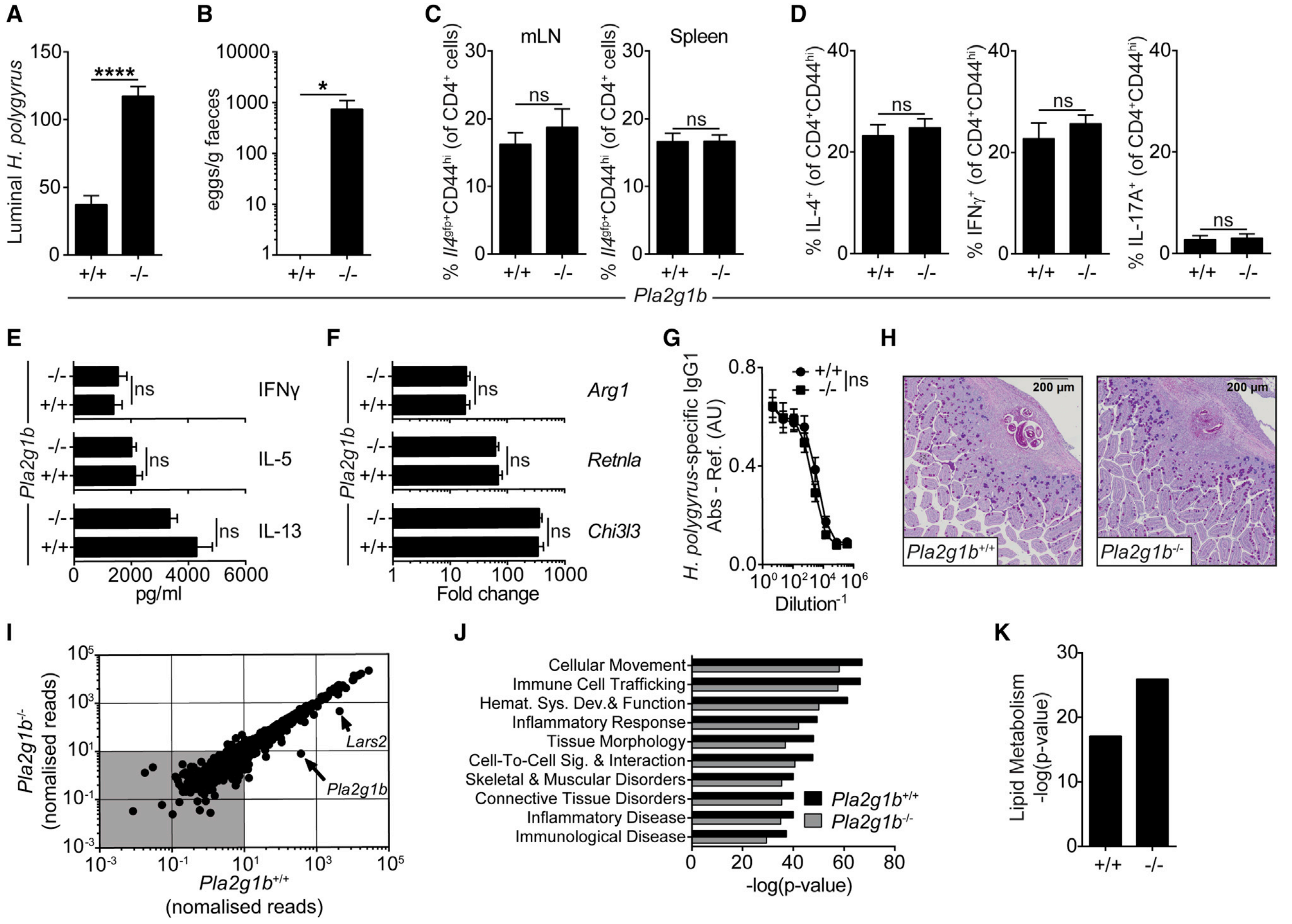

Figure 2. Type 2 Immunity Is Insufficient for Resistance to Intestinal Helminth Infection in the Absence of *Pla2g1b*

(A) Luminal *H.p.* worms in the small intestine 14 days after 2° infection.

(B) Fecal egg counts 14 days after 2° *H.p.* infection.

(C) Frequency of *Il4*$^{gfp+}$CD44^{hi} CD4$^+$ cells in the mesenteric lymph node (mLN) and the spleen of mice 7 days after 2° infection.

(D) Frequency of cytokine$^+$ CD4$^+$CD44^{hi} cells in the mLN of mice 7 days after 2° infection.

(E) *ex vivo H.p.* antigen-specific cytokine production from the mLN of mice 7 days after 2° infection.

(F) Gene expression in the small intestine of mice 7 days after 2° *H.p.* infection.

(G) *H.p.* antigen-specific IgG1 in the serum from mice 7 days after 2° *H.p.* infection.

(H) Mucus staining (Alcian blue-periodic acid-Schiff) of the small intestine from mice 7 days after 2° *H.p.* infection.

(I) RNA-sequencing-generated transcriptional landscape of the small intestine of WT and *Pla2g1b*$^{-/-}$ mice 7 days after 2° *H.p.* infection.

(J) Top 10 pathways predicted to be activated 7 days after 2° *H.p.* infection (relative to strain-naive, 2-fold filter, $p < 0.05$).

(K) Lipid metabolism pathway predicted activation score 7 days after 2° *H.p.* infection (relative to strain-naive, 2-fold filter, $p < 0.05$).

Data are represented as mean ± SEM, n = 5–6, representative of at least three independent experiments; ns = not significant, * = $p < 0.05$, **** = $p < 0.0001$ determined using a two-way ANOVA with Sidak's multiple comparison analysis or an unpaired two-tailed t test. See also Figures S2–S4.

Pla2g1b$^{-/-}$ mice failing to expel a 2° *H.p.* infection and retaining a patent infection (Figures 2A and 2B). The absence of PLA$_2$g1B did not affect the expression of other detectable PLA$_2$ family members (Figure S1E). PLA$_2$g1b was also required for expulsion of *N. brasiliensis*, which also infects the small intestine (Figure S2A); however, *Pla2g1b* was not required for expulsion of the cecum-dwelling whipworm *Trichuris muris* (Figure S2B).

IL-4 and type 2 immune responses orchestrate many of the known anti-helminth, immune-driven expulsion pathways (Hashimoto et al., 2009). We therefore assessed innate and adaptive type 2 immune responses in *Pla2g1b*$^{-/-}$ mice and, to our surprise, found that all measured type 2 immune responses were intact. Specifically, by crossing *Pla2g1b*$^{-/-}$ mice onto an *Il4*^{gfp} reporter background or by measuring IL-4 protein by intra-cellular staining, we found that Th2 cell commitment and differentiation were equivalent between genotypes (Figures 2C and 2D). Parasite-specific, Th2-derived cytokines IL-5 and IL-13 were also comparable (Figure 2E), with no measurable difference in IFNγ$^+$ or IL-17A$^+$ T cells (Figure 2D). Lymphocyte populations in *Pla2g1b*$^{-/-}$ mice, both at baseline and following 2° *H.p.* infection, were comparable to WT mice, including both CD4$^+$ and CD8$^+$ T cells, ILC2s, which support early Th2 differentiation (Pelly et al., 2016) and regulatory T cells, which inhibit type 2 immune responses (Wilson et al., 2005; Figures S2C–S2J and S3A).

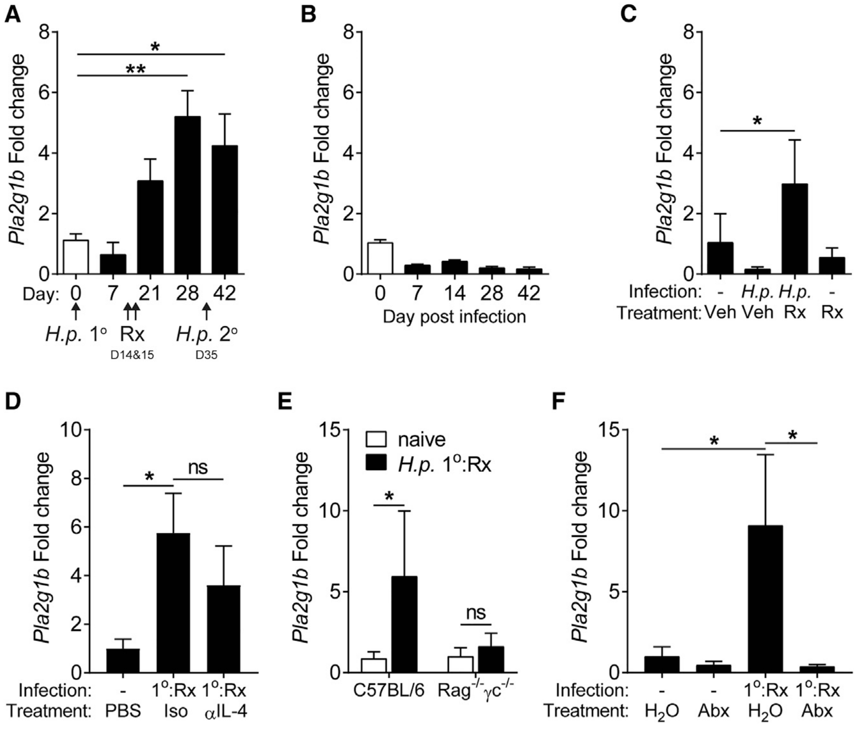

Figure 3. Intestinal *Pla2g1b* Is Regulated by the Microbiota and Rag- and Common Gamma Chain-Dependent Cells in Resistant Mice

(A) Kinetics of *Pla2g1b* expression in the small intestine during *H.p.* 2° infection model.

(B) Kinetics of *Pla2g1b* expression in the small intestine during *H.p.* 1° infection, n = 6.

(C) *Pla2g1b* expression at day 28 (14 days post-Rx) in the small intestine, n = 5.

(D) *Pla2g1b* expression at day 28 (14 days post-Rx, 1°-Rx) in the small intestine following anti-IL-4 (αIL-4) or Isotype (Iso) treatment, n = 9–10 (data pooled from two independent experiments).

(E) *Pla2g1b* expression at day 28 (14 days post-Rx, 1° Rx) in the small intestine, n = 7–10 (data pooled from two independent experiments).

(F) *Pla2g1b* expression at day 28 (14 days post-Rx, 1° Rx) in the small intestine following antibiotic treatment, n = 9–10 (data pooled from two independent experiments).

Data are represented as mean ± SEM, n = 3. All data are representative of at least two independent experiments; * = $p < 0.05$, ** = $p < 0.01$, determined using a one-way ANOVA with Dunnett's multiple comparison analysis, unpaired t test, or a Mann-Whitney test.

Alternatively activated macrophage-associated genes, which are also essential for immunity to *H.p.* (Anthony et al., 2006), were similar between genotypes in vivo (Figure 2F) and following polarization in vitro (Figure S3B). Finally, B cell frequencies and serum antibodies, including *H.p.*-specific IgG1 and IgE, which are important for preventing adult worm development (McCoy et al., 2008), were comparable between WT and *Pla2g1b*$^{-/-}$ mice (Figures 2G, S3C, and S3D). Physiological responses—including goblet cell hyperplasia and mucus hypersecretion, which correlate with expulsion—were also comparable between WT and *Pla2g1b*$^{-/-}$ mice (Figures 2H and S3E). RNA sequencing and pathway analysis of the transcriptome of duodenal tissue from naive and infected *Pla2g1b*$^{-/-}$ mice 7 days after 2° *H.p.* infection identified that the transcriptional responses and associated pathways were very similar to WT mice with the exception of 2 genes, *Pla2g1b* and *Lars2* (Figures 2I, 2J, and S3F). To our surprise, predicted activation of lipid metabolism pathways and synthesis of bioactive lipids, including cysteine leukotrienes and prostaglandin E 2 (PGE_2), serum fatty acid metabolites, and serum Lysophosphatidylcholines (LPC), were unaffected in *Pla2g1b*$^{-/-}$ mice (Figures 2K and S3G–S3I), most likely due to unaltered expression of other phospholipase A_2 enzymes (Figure S1E). These data suggest that the failure to expel *H.p.* in *Pla2g1b*$^{-/-}$ mice was not due to altered lipid metabolism or known immune-mediated or stromal-associated expulsion mechanisms.

To determine the source of PLA_2g1B and its regulation, we monitored *Pla2g1b* expression in duodenal tissue following 1° infection, 1° infection and drug cure, and 2° infection. *Pla2g1b* was significantly upregulated 14 days after drug cure of a 1° infection (day 28) and was maintained with or without a 2° infection (Figures 3A and 1F). This correlated with the development and maintenance of resistance to 2° infection. Despite a strong type 2 immune response, *Pla2g1b* expression was not upregulated in mice given a 1° infection (Figure 3B) or following drug treatment alone (Figure 3C), but had a trend to be reduced. We hypothesize that a 1° *H.p.* infection may actively inhibit PLA_2g1B through secretion of immunomodulatory proteins. However, this remains to be tested. IL-4 is essential for resistance to a 2° *H.p.* infection (Urban et al., 1991b). We therefore investigated whether IL-4 was required for upregulation of *Pla2g1b* in resistant mice. Anti-IL-4 antibody treatment prior to and during drug-treatment did not prevent *Pla2g1b* upregulation in resistant mice (Figure 3D), suggesting that *Pla2g1b* is not regulated in an IL-4 or type-2 immune dependent manner. To test whether Rag-dependent, adaptive immune cells and common gamma chain (cg)-dependent innate immune cells and signaling were required for *Pla2g1b* expression, we infected and drug-treated WT and *Rag2*$^{-/-}$*cg*$^{-/-}$ mice. Following drug treatment, WT, but not *Rag2*$^{-/-}$*cg*$^{-/-}$, mice upregulated *Pla2g1b*, indicating that at this time prior to immune-mediated active expulsion, adaptive and cg-dependent signaling was required for the upregulation of *Pla2g1b in vivo* (Figure 3E).

To identify additional type 2 independent mechanisms of *Pla2g1b* upregulation, we tested whether the host microbiota contributed to *Pla2g1b* regulation. Antibiotic-treated mice completely failed to upregulate *Pla2g1b* (Figure 3F), indicating that intestinal microbiota are essential for elevated *Pla2g1b* expression. Intestinal microbiota changes following helminth infection (Giacomin et al., 2016; Rausch et al., 2013; Reynolds et al., 2014; Zaiss et al., 2015) have previously been reported. However, whether a microbiota-driven *Pla2g1b* axis has evolved to restore intestinal homeostasis and protect from small-intestine dwelling helminths is unclear.

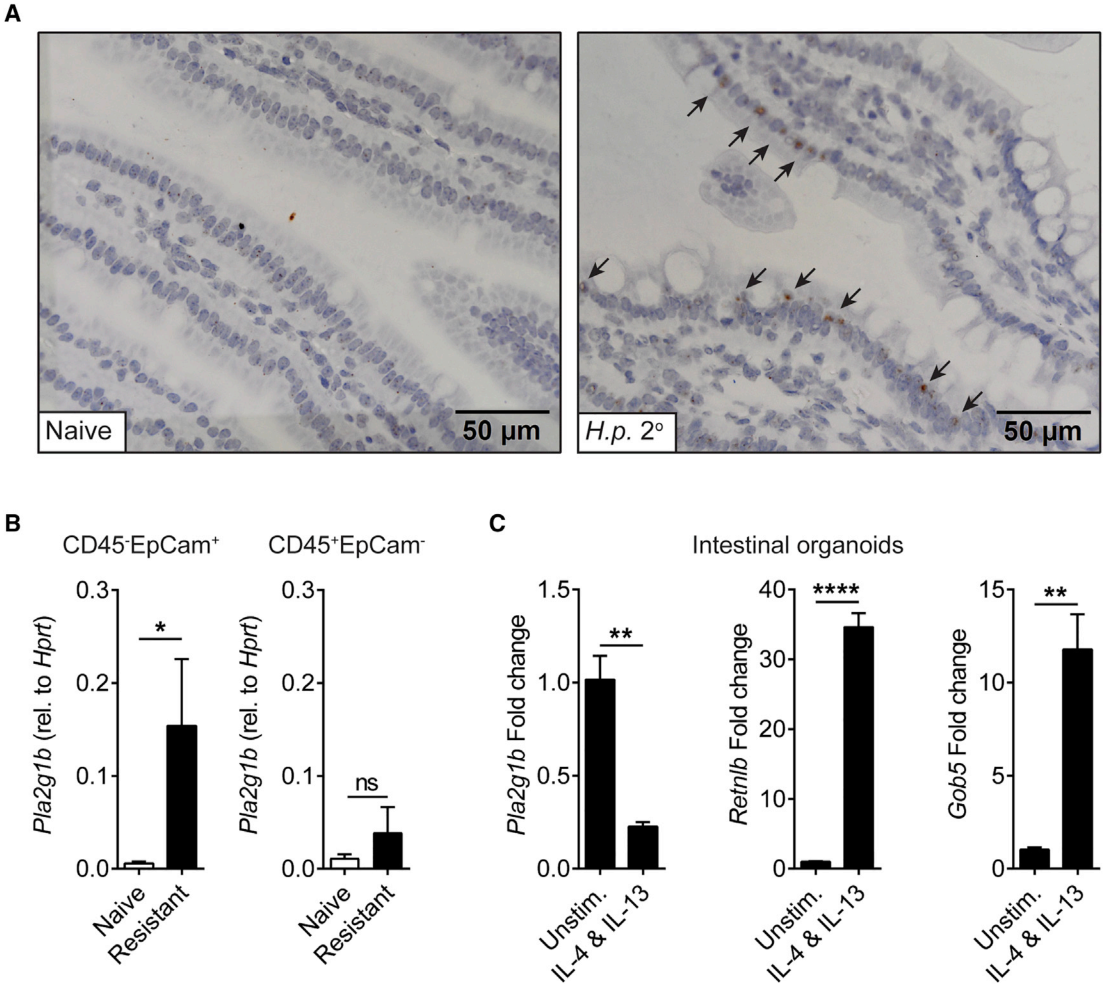

Figure 4. Epithelial-Cell-Derived *Pla2g1b* Is Negatively Regulated by IL-4Rα-Signaling

(A) *Pla2g1b* detection by RNAScope ISH in the small intestine 7 days after 2° *H.p.* infection.

(B) *Pla2g1b* expression in fluorescence-activated, cell sorted (FACS) $CD45^-EpCam^+$ and $CD45^+EpCam^-$ cells from the intestinal epithelium from naive and resistant (day 28) mice, n = 6.

(C) *Pla2g1b, Retnlb*, and *Gob5* expression in intestinal organoid cultures following stimulation with rIL-4 and rIL-13.

Data are represented as mean ± SEM, n = 3. All data are representative of at least two independent experiments. * = $p < 0.05$, ** = $p < 0.01$, **** = $p < 0.0001$ determined using an unpaired t test or a Mann-Whitney test. See also Figure S5.

In situ hybridization localized *Pla2g1b* expression to the epithelial layer following *H.p.* 2° infection (Figures 4A and S4A) rather than in the granuloma where larvae embed (Figure S4B). We confirmed that *Pla2g1b* was elevated in FACS-purified $CD45^-EpCam^+$ epithelial cells rather than $CD45^+EpCam^-$ cells isolated from drug-cured mice compared to naive (Figure 4B). With the recent identification of tuft cells as important in anti-helminth immunity (Gerbe et al., 2016; Howitt et al., 2016; von Moltke et al., 2016), we analyzed the expression of tuft-cell-specific markers in our RNA sequencing data from the *H.p.* challenge infection model (Figure 1A). We identified significant upregulation of the tuft cell markers *Dclk*, *Trpm5*, *Siglec5* and *Pou2f3* in resistant *H.p.* 2° infected, but not in *H.p.* 1° infected mice (Figure S4C). Importantly, expression of tuft cell markers, which peaked transiently following infection, did not correlate with *Pla2g1b* expression, which was upregulated following drug cure of 1° infection and maintained with or without 2° challenge infection. These data suggest that *Pla2g1b* expression was not restricted to tuft cells, or at least tuft-cell-associated gene expression. In addition, although *Pla2g1b* is abundantly expressed in the pancreas (Eerola et al., 2006), it was not differentially expressed in the pancreas following infection, drug treatment, or reinfection (Figure S4D). Both our findings (Figures 2 and S3) and previous data (Hollie and Hui, 2011; Labonté et al., 2010) suggest that PLA_2g1B does not contribute to altered dietary phospholipid digestion at steady state or during *H.p.* infection. To determine how *Pla2g1b* was regulated in epithelial cells, we generated *ex vivo* organoid cultures (Sato et al., 2009) and found that IL-4R signaling decreased *Pla2g1b* expression but increased the expulsion-related genes Relmβ (*Retnlb*) and *Gob5* (Hashimoto et al., 2009; Herbert et al., 2009; Figure 4C). These data again uncouple *Pla2g1b* expression from type 2

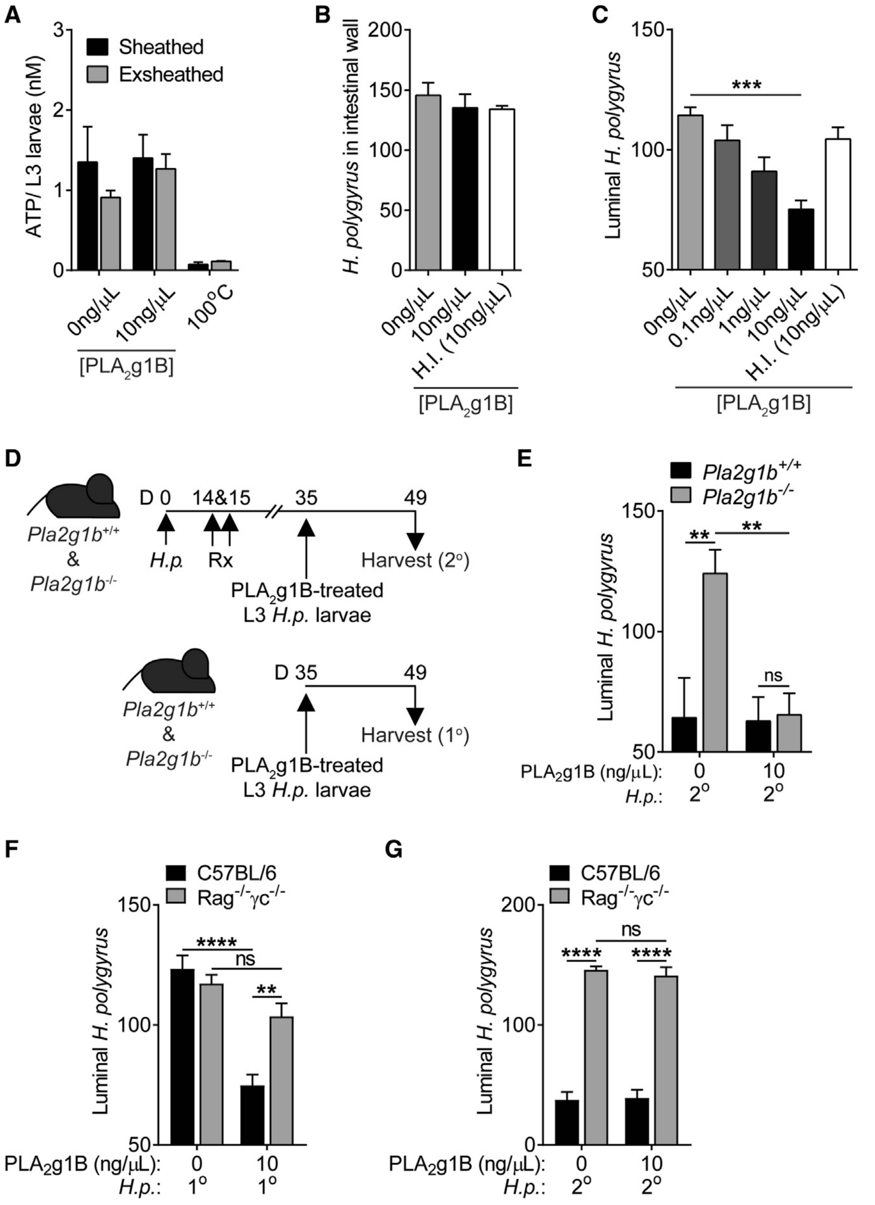

Figure 5. PLA$_2$g1B Has Direct Anthelmintic Properties

(A) ATP concentration of *H.p.* L3 larvae after 24 hr treatment with PLA$_2$g1B, n = 3.

(B) Number of *H.p.* larvae imbedded in the small intestinal wall 5 days after infection following 24 hr treatment with PLA$_2$g1B, n = 5.

(C) Luminal *H.p.* worms in the small intestine 14 days after 1° infection following 24 hr treatment with PLA$_2$g1B, n = 10 (data pooled from two independent experiments).

(D) *Pla2g1b*$^{-/-}$ or WT mice were orally infected with 200 L3 *H.p.* larvae on day 0 and were drug treated (Rx) on days 14 and 15. Mice were then reinfected with PLA$_2$g1B-treated L3 *H.p.* larvae on day 35 and harvested 14 days after infection (2°). Another cohort of *Pla2g1b*$^{-/-}$ or WT mice were orally infected with 200 L3 *H.p.* larvae on day 35 and harvested 14 days after infection (1°).

(E) Luminal *H.p.* worms in the small intestine 14 days after 1° or 2° infection following 24 hr treatment with PLA$_2$g1B.

(F) Luminal *H.p.* worms in the small intestine 14 days after 1° infection following 24 hr treatment with PLA$_2$g1B.

(G) Luminal *H.p.* worms in the small intestine 14 days after 2° infection following 24 hr treatment with PLA$_2$g1B.

Data are represented as mean ± SEM, n = 4–5. All data re representative of at least two independent experiments. * = $p < 0.05$, *** = $p < 0.001$, **** = $p < 0.0001$ determined using a two-way ANOVA with Sidak's multiple comparison analysis, one-way ANOVA with Dunnett's multiple comparison analysis, or an unpaired two-tailed t test. See also Figure S6.

immune pathways that drive expulsion mechanisms (Anthony et al., 2006; Herbert et al., 2009; Urban et al., 1991b) and suggest that type 2 immunity may negatively regulate *Pla2g1b* expression to protect host tissues from the potent effects of phospholipase enzymes (Murakami et al., 2011).

Other sPLA$_2$ family members can degrade bacterial membranes and protect from fungal infections (Balestrieri et al., 2009; Degousee et al., 2002; Koduri et al., 2002; Weinrauch et al., 1998). We therefore asked whether PLA$_2$g1B had a direct effect on *H.p.* by treating sheathed or exsheathed L3 *H.p.* larvae (to recapitulate the status of the larvae in the small intestine after passing through the stomach) (Sommerville and Bailey, 1973) and adult *H.p.* worms with recombinant PLA$_2$g1B. No effect was identified on the fitness of treated L3 larvae, isolated L4 larvae, or adult worms, as determined by ATP concentration (Figures 5A, S5A, and S5B). To test the infectivity and viability of PLA$_2$g1B-treated L3 larvae, we infected mice with PLA$_2$g1B-treated L3 larvae and found that PLA$_2$g1B-treated L3 larvae could embed into the intestinal wall (Figure 5B) However, significantly fewer treated larvae developed into adulthood (Figure 5C), impacting egg recovery in the feces, although failing to reach statistical significance (Figure S5C). PLA$_2$g1B-mediated effects on L3 larvae were dependent on the catalytic activity of PLA$_2$g1B as the PLA$_2$ irreversible inhibitor manoalide completely abrogated the protective effect of PLA$_2$g1B treatment *in vitro* (Figure S5D).

The protective effect of PLA$_2$g1B-treated L3 larvae during a 1° infection did not recapitulate the full killing, expulsion, and clearance of worms observed during 2° *H.p.* infection (Figure 2). We therefore hypothesized that, for clearance of *H.p.*, a combined PLA$_2$g1B-mediated impact on larvae in concert with immune-mediated physiological responses would be required. To test this, we infected and drug cured WT and *Pla2g1b*$^{-/-}$ mice to elicit robust type 2 immune responses (Figure 2) and challenged mice with vehicle- or PLA$_2$g1B-treated L3 larvae (model, Figure 5D). WT mice cleared the majority of either vehicle or PLA$_2$g1B-treated *H.p.* larvae. *Pla2g1b*$^{-/-}$ mice failed to clear vehicle-treated L3 larvae, as expected (Figure 2). However,

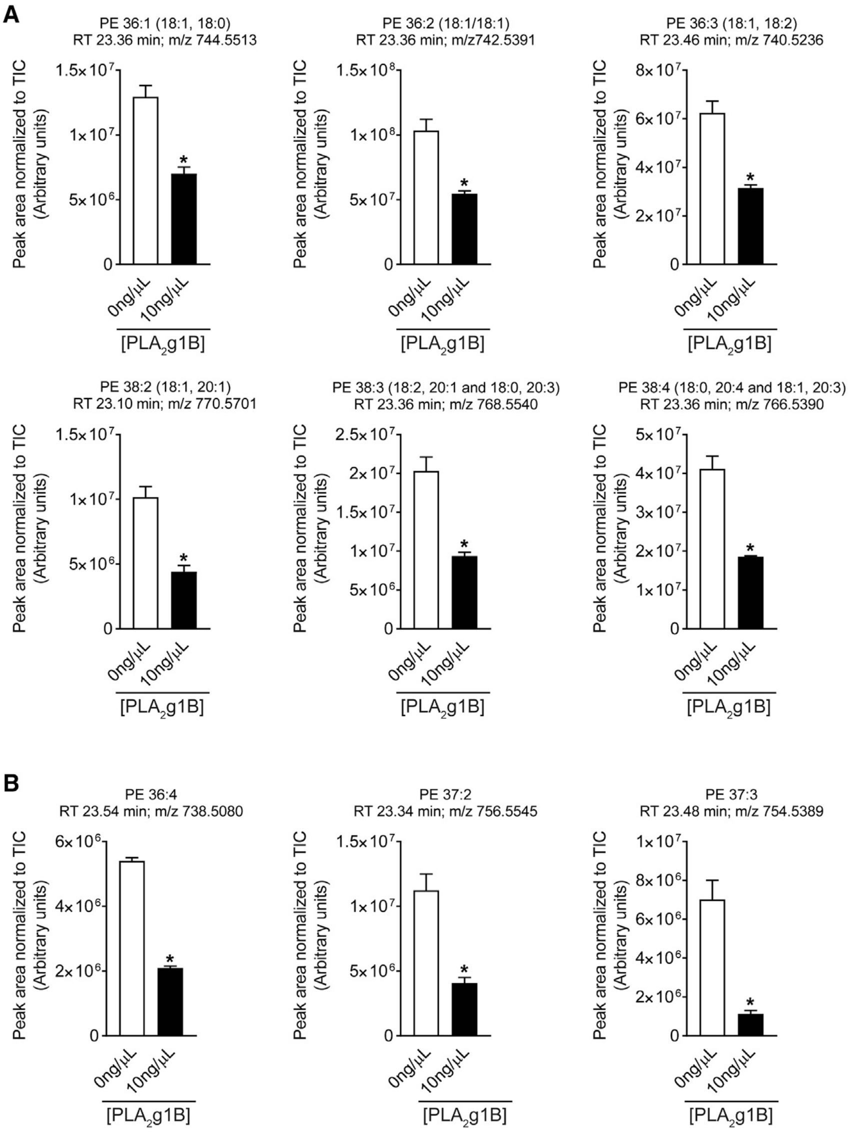

Figure 6. Pla2g1b-Treatment Related Changes in Lipid Abundance

Relative abundances of phosphatidylethanolamine (PE) and other lipids extracted from PLA_2g1B-treated (10 ng/μL) and control-treated (0 ng/μL) larvae.

(A) Identified PEs. Features were regarded as "identified" by comparison of their precursor ion and MS/MS fragments with the LipidBlast library, as outlined in Figure S1. The arrangement of fatty acid moieties on the glycerol backbone (i.e., whether in the *sn*-1 or *sn*-2 position) and the position of double bonds could not be inferred. PE 38:3 and PE 38:4 were detected as a mixture of different fatty acid moieties.

(B) Putatively identified (annotated) PEs. The features could be "annotated" as PEs by comparison of peak retention time and inter-cluster mass shifts of 28 Da (CH_2CH_2) and intra-cluster mass shifts of 2 Da (indicative of difference in double bond number [fatty acid saturation]) with other, identified PEs. MS/MS could not be performed due to low abundance. Data are shown as normalized intensities expressed in arbitrary units. Data are represented as mean ± SEM, n = 3. * = $p < 0.05$. TIC: Total ion current. See also Figure S7.

H.p. killing, expulsion, and clearance were completely rescued in *Pla2g1b*$^{-/-}$ mice when L3 larvae were directly treated with PLA_2g1B (Figure 5E), suggesting that killing and expulsion of *H.p.* requires both PLA_2g1B-mediated effects on L3 larvae and immune-mediated expulsion mechanisms.

The requirement for a combined functional immune compartment alongside direct PLA_2g1B-mediated action was confirmed by infecting *Rag*$^{-/-}$*cg*$^{-/-}$ mice with PLA_2g1B-treated larvae. Upon 1° infection, *Rag*$^{-/-}$*cg*$^{-/-}$ mice fail to expel PLA_2g1B-treated larvae, unlike WT mice (Figure 5F) These data highlight the clear requirement of a competent immune compartment for parasite expulsion. This observation was reinforced when *Rag*$^{-/-}$*cg*$^{-/-}$ mice that were infected, drug cured, and challenged with PLA_2g1B-treated L3 larvae failed to expel *H.p.*, unlike WT mice (Figure 5G). It is important to note that, under these 2° challenge conditions in *Rag*$^{-/-}$*cg*$^{-/-}$ mice, we cannot distinguish the requirement of *Rag*$^{-/-}$*cg*$^{-/-}$-driven *Pla2g1b* expression (required for parasite killing) from *Rag*$^{-/-}$*cg*$^{-/-}$-dependent immune responses (required for expulsion).

Nevertheless, these data demonstrate that PLA_2g1B has direct anthelminthic properties distinct from type 2 immune responses and that PLA_2g1B is essential for intestinal helminth clearance during 2° *H.p.* infection. Furthermore, these data add to other discovered anti-microbial properties of $sPLA_2$ enzymes (Balestrieri et al., 2009; Degousee et al., 2002; Koduri et al., 2002; Maizels and Hewitson, 2016; Weinrauch et al., 1998) and to the site-specific arsenal of anti-microbial responses in the small intestine (Gallo and Hooper, 2012).

Finally, to identify the direct effects of PLA_2g1B on *H.p.* L3 larvae, we treated larvae for 24 hr with PLA_2g1B or control buffer and subjected the treated larvae to scanning electron microscopy (SEM) and lipid composition analysis using liquid chromatography-mass spectrometry (LC-MS). Although SEM did not reveal any structural changes or alterations in membrane integrity (Figure S5E), LC-MS analysis identified a significantly lower phospholipid abundance in the PLA_2g1B-treated larvae when compared to untreated controls (Figure S6A). Of the 1,165 apolar features detected, only 112 were significantly different following PLA_2g1B treatment; 6 were identified as phosphatidylethanolamines (PEs) (by comparing their precursor ion and MS/MS fragments with the LipidBlast library) (Figures 6A and S6A–S6E), with each being of lower abundance in the treated larvae. We also identified a similar trend in a number of putatively identified PEs (as determined by comparison of peak retention time with other identified PEs, together with intercluster mass shifts of 28 Da (CH_2CH_2) and intracluster mass shifts of 2 Da, indicative of differences in double-bond number, i.e., saturation)

(Figure 6B). The remaining, significantly different apolar features were seen to be both increased and decreased following PLA_2g1B treatment; however, these features were unable to be identified (examples shown in Figure S6F). PEs are highly abundant phospholipids found in membranes of bacteria, yeast, and mammals and are required for an array of cellular functions including membrane fusion, cytokinesis, cell division, membrane curvature, and as a substrate for subsequent products (Wellner et al., 2013). The relative reduction in PEs in PLA_2g1B-treated *H.p.* L3 larvae provides one explanation for many putative downstream impacts on larval integrity, health, and infectivity. For example, studies in yeast and *Caenorhabditis elegans* identified that low levels of PE can cause ER stress and disrupt vesicle trafficking (Wang et al., 2014). Whether similar effects are observed in PLA_2g1B-treated *H.p.* are currently unclear. Another possible explanation is that reducing larval phospholipids may allow for greater immune cell recognition and larval trapping in the tissue (Anthony et al., 2006; Esser-von Bieren et al., 2015), although this has not been tested here.

Taken together, our data uncouple type 2 immune-mediated anti-helminth expulsion mechanisms from the production of host enzymes that mediate direct anti-helminth activity, which are regulated by the microbiota and require competent innate and adaptive immunity. Specifically, we highlight a previously overlooked role for epithelial-cell-derived PLA_2g1B as an essential endogenous anthelmintic that has direct effects on invading larvae, possibly by reducing phospholipid levels. Identifying mechanisms that regulate site-specific expression of *Pla2g1b* and the functional role of phospholipids in helminths may provide avenues toward greater protection from helminth infection.

STAR★METHODS

Detailed methods are provided in the online version of this paper and include the following:

- KEY RESOURCES TABLE
- CONTACT FOR REAGENT AND RESOURCE SHARING
- EXPERIMENTAL MODEL AND SUBJECT DETAILS
  - Animal Strains
  - Organoid Culture
  - Bone Marrow-derived Macrophage (BMDM) Culture and Stimulation
- METHOD DETAILS
  - Parasite Infections and PLA_2g1B Treatment
  - ATP Assay
  - Antibiotic Treatment
  - Antibody Treatment
  - Histology, In Situ Hybridization
  - RNA Sequencing and Analysis
  - Quantitative Real-time Polymerase Chain Reaction
  - PLA_2 Activity Assay
  - Cell Isolation
  - Flow Cytometry and Cell Sorting
  - Ex Vivo Stimulations
  - ELISAs
  - Scanning Electron Microscopy (SEM)
  - Serum Chemistry
  - Lipid Extraction and Analysis
  - Experimental Design
- QUANTIFICATION AND STATISTICAL ANALYSIS
- DATA AND SOFTWARE AVAILABILITY

SUPPLEMENTAL INFORMATION

Supplemental Information includes six figures and four tables and can be found with this article online at https://doi.org/10.1016/j.chom.2017.09.006.

AUTHOR CONTRIBUTIONS

L.J.E. performed and analyzed the majority of experiments. V.S.P., S.M.C., J.P.-L., S.C., and Y.K. assisted with mouse studies and flow cytometry experiments; A.S. contributed to the design of the RNA sequencing experimental design; N.N. assisted with the analysis of the RNA sequencing data; and A.M. assisted with organoid experiments. L.C. assisted with SEM experiments. M.S.d.S. and J.I.M. performed and analyzed LC-MS experiments. H.H. kindly provided *N. brasiliensis* L3 larvae, and D.Y.H. donated *Pla2g1b*$^{-/-}$ mice. L.J.E. and M.S.W. designed experiments and wrote the manuscript.

ACKNOWLEDGMENTS

The authors thank The Francis Crick Institute Advanced Sequencing Facility, especially Abdul Sesay, Leena Bhaw, and Harsha Jani, for the RNA sequencing critical to this work. We would like to thank The Francis Crick Institute Flow Cytometry facility (Bhavik Patel, Graham Preece, Wayne Turnbull, and Phil Hobson) for cell sorting services and Gitta Stockinger for critically reading the manuscript. We are indebted to The Francis Crick Institute Procedural Service Section for the production of GA lines; to Biological Services, especially Trisha Norton, Keith Williams, and Adebambo Adekoya, for animal husbandry and technical support; to Radma Mahmood, Radika Anand and Bradley Spencer-Dene for help with histology and *in situ hybridisation*; and to Riccardo Guidi for constructive discussions and technical assistance. This work was supported by the Francis Crick Institute (grant number FCI01) which receives its core funding from Cancer Research UK, the UK Medical Research Council (MC_UP_A253_1028), and the Wellcome Trust. D.Y.H. was supported by NIH grant RO1 DK112657. M.S.W. is an employee of Genentech Inc.

Received: January 29, 2017
Revised: July 5, 2017
Accepted: September 14, 2017
Published: October 11, 2017

REFERENCES

Allen, J.E., and Maizels, R.M. (2011). Diversity and dialogue in immunity to helminths. Nat. Rev. Immunol. *11*, 375–388.

Anthony, R.M., Urban, J.F., Jr., Alem, F., Hamed, H.A., Rozo, C.T., Boucher, J.L., Van Rooijen, N., and Gause, W.C. (2006). Memory T(H)2 cells induce alternatively activated macrophages to mediate protection against nematode parasites. Nat. Med. *12*, 955–960.

Anthony, R.M., Rutitzky, L.I., Urban, J.F., Jr., Stadecker, M.J., and Gause, W.C. (2007). Protective immune mechanisms in helminth infection. Nat. Rev. Immunol. *7*, 975–987.

Balestrieri, B., Maekawa, A., Xing, W., Gelb, M.H., Katz, H.R., and Arm, J.P. (2009). Group V secretory phospholipase A2 modulates phagosome maturation and regulates the innate immune response against Candida albicans. J. Immunol. *182*, 4891–4898.

Camberis, M., Le Gros, G., and Urban, J., Jr. (2003). Animal Model of Nippostrongylus brasiliensis and Heligmosomoides polygyrus. Curr. Protoc. Immunol. *Chapter 19*. Unit 19.12.

Degousee, N., Ghomashchi, F., Stefanski, E., Singer, A., Smart, B.P., Borregaard, N., Reithmeier, R., Lindsay, T.F., Lichtenberger, C., Reinisch,

W., et al. (2002). Groups IV, V, and X phospholipases A2s in human neutrophils: role in eicosanoid production and gram-negative bacterial phospholipid hydrolysis. J. Biol. Chem. *277*, 5061–5073.

Eerola, L.I., Surrel, F., Nevalainen, T.J., Gelb, M.H., Lambeau, G., and Laine, V.J. (2006). Analysis of expression of secreted phospholipases A2 in mouse tissues at protein and mRNA levels. Biochim. Biophys. Acta *1761*, 745–756.

Esser-von Bieren, J., Volpe, B., Kulagin, M., Sutherland, D.B., Guiet, R., Seitz, A., Marsland, B.J., Verbeek, J.S., and Harris, N.L. (2015). Antibody-mediated trapping of helminth larvae requires CD11b and Fcγ receptor I. J. Immunol. *194*, 1154–1163.

Finkelman, F.D., Shea-Donohue, T., Goldhill, J., Sullivan, C.A., Morris, S.C., Madden, K.B., Gause, W.C., and Urban, J.F., Jr. (1997). Cytokine regulation of host defense against parasitic gastrointestinal nematodes: lessons from studies with rodent models. Annu. Rev. Immunol. *15*, 505–533.

Gallo, R.L., and Hooper, L.V. (2012). Epithelial antimicrobial defence of the skin and intestine. Nat. Rev. Immunol. *12*, 503–516.

Gerbe, F., Sidot, E., Smyth, D.J., Ohmoto, M., Matsumoto, I., Dardalhon, V., Cesses, P., Garnier, L., Pouzolles, M., Brulin, B., et al. (2016). Intestinal epithelial tuft cells initiate type 2 mucosal immunity to helminth parasites. Nature *529*, 226–230.

Giacomin, P., Zakrzewski, M., Jenkins, T.P., Su, X., Al-Hallaf, R., Croese, J., de Vries, S., Grant, A., Mitreva, M., Loukas, A., et al. (2016). Changes in duodenal tissue-associated microbiota following hookworm infection and consecutive gluten challenges in humans with coeliac disease. Sci. Rep. *6*, 36797.

Grencis, R.K., Hültner, L., and Else, K.J. (1991). Host protective immunity to Trichinella spiralis in mice: activation of Th cell subsets and lymphokine secretion in mice expressing different response phenotypes. Immunology *74*, 329–332.

Hashimoto, K., Uchikawa, R., Tegoshi, T., Takeda, K., Yamada, M., and Arizono, N. (2009). Depleted intestinal goblet cells and severe pathological changes in SCID mice infected with Heligmosomoides polygyrus. Parasite Immunol. *31*, 457–465.

Hasnain, S.Z., Wang, H., Ghia, J.E., Haq, N., Deng, Y., Velcich, A., Grencis, R.K., Thornton, D.J., and Khan, W.I. (2010). Mucin gene deficiency in mice impairs host resistance to an enteric parasitic infection. Gastroenterology *138*, 1763–1771.

Hasnain, S.Z., Evans, C.M., Roy, M., Gallagher, A.L., Kindrachuk, K.N., Barron, L., Dickey, B.F., Wilson, M.S., Wynn, T.A., Grencis, R.K., and Thornton, D.J. (2011). Muc5ac: a critical component mediating the rejection of enteric nematodes. J. Exp. Med. *208*, 893–900.

Herbert, D.R., Yang, J.Q., Hogan, S.P., Groschwitz, K., Khodoun, M., Munitz, A., Orekov, T., Perkins, C., Wang, Q., Brombacher, F., et al. (2009). Intestinal epithelial cell secretion of RELM-β protects against gastrointestinal worm infection. J. Exp. Med. *206*, 2947–2957.

Hollie, N.I., and Hui, D.Y. (2011). Group 1B phospholipase A_2 deficiency protects against diet-induced hyperlipidemia in mice. J. Lipid Res. *52*, 2005–2011.

Hotez, P.J., Brindley, P.J., Bethony, J.M., King, C.H., Pearce, E.J., and Jacobson, J. (2008). Helminth infections: the great neglected tropical diseases. J. Clin. Invest. *118*, 1311–1321.

Howitt, M.R., Lavoie, S., Michaud, M., Blum, A.M., Tran, S.V., Weinstock, J.V., Gallini, C.A., Redding, K., Margolskee, R.F., Osborne, L.C., et al. (2016). Tuft cells, taste-chemosensory cells, orchestrate parasite type 2 immunity in the gut. Science *351*, 1329–1333.

Katona, I.M., Urban, J.F., Jr., Kang, S.S., Paul, W.E., and Finkelman, F.D. (1991). IL-4 requirements for the generation of secondary in vivo IgE responses. J. Immunol. *146*, 4215–4221.

Koduri, R.S., Grönroos, J.O., Laine, V.J., Le Calvez, C., Lambeau, G., Nevalainen, T.J., and Gelb, M.H. (2002). Bactericidal properties of human and murine groups I, II, V, X, and XII secreted phospholipases A(2). J. Biol. Chem. *277*, 5849–5857.

Labonté, E.D., Kirby, R.J., Schildmeyer, N.M., Cannon, A.M., Huggins, K.W., and Hui, D.Y. (2006). Group 1B phospholipase A2-mediated lysophospholipid absorption directly contributes to postprandial hyperglycemia. Diabetes *55*, 935–941.

Labonté, E.D., Pfluger, P.T., Cash, J.G., Kuhel, D.G., Roja, J.C., Magness, D.P., Jandacek, R.J., Tschöp, M.H., and Hui, D.Y. (2010). Postprandial lysophospholipid suppresses hepatic fatty acid oxidation: the molecular link between group 1B phospholipase A2 and diet-induced obesity. FASEB J. *24*, 2516–2524.

Maizels, R.M., and Hewitson, J.P. (2016). Myeloid cell phenotypes in susceptibility and resistance to helminth parasite infections. Microbiol. Spectr. *4*, https://doi.org/10.1128/microbiolspec.MCHD-0043-2016.

Maizels, R.M., Hewitson, J.P., Murray, J., Harcus, Y.M., Dayer, B., Filbey, K.J., Grainger, J.R., McSorley, H.J., Reynolds, L.A., and Smith, K.A. (2012). Immune modulation and modulators in Heligmosomoides polygyrus infection. Exp. Parasitol. *132*, 76–89.

McCoy, K.D., Stoel, M., Stettler, R., Merky, P., Fink, K., Senn, B.M., Schaer, C., Massacand, J., Odermatt, B., Oettgen, H.C., et al. (2008). Polyclonal and specific antibodies mediate protective immunity against enteric helminth infection. Cell Host Microbe *4*, 362–373.

Meyer, F., Kimura, S., and Mueller, J.F. (1966). Lipid metabolism in the larval and adult forms of the tapeworm Spirometra mansonoides. J. Biol. Chem. *241*, 4224–4232.

Mohrs, M., Shinkai, K., Mohrs, K., and Locksley, R.M. (2001). Analysis of type 2 immunity in vivo with a bicistronic IL-4 reporter. Immunity *15*, 303–311.

Murakami, M., Taketomi, Y., Miki, Y., Sato, H., Hirabayashi, T., and Yamamoto, K. (2011). Recent progress in phospholipase A_2 research: from cells to animals to humans. Prog. Lipid Res. *50*, 152–192.

Murakami, M., Yamamoto, K., Miki, Y., Murase, R., Sato, H., and Taketomi, Y. (2016). The roles of the secreted phospholipase A2 gene family in immunology. Adv. Immunol. *132*, 91–134.

Pelly, V.S., Kannan, Y., Coomes, S.M., Entwistle, L.J., Rückerl, D., Seddon, B., MacDonald, A.S., McKenzie, A., and Wilson, M.S. (2016). IL-4-producing ILC2s are required for the differentiation of TH2 cells following Heligmosomoides polygyrus infection. Mucosal Immunol. *9*, 1407–1417.

Rausch, S., Held, J., Fischer, A., Heimesaat, M.M., Kühl, A.A., Bereswill, S., and Hartmann, S. (2013). Small intestinal nematode infection of mice is associated with increased enterobacterial loads alongside the intestinal tract. PLoS One *8*, e74026.

Reynolds, L.A., Filbey, K.J., and Maizels, R.M. (2012). Immunity to the model intestinal helminth parasite Heligmosomoides polygyrus. Semin. Immunopathol. *34*, 829–846.

Reynolds, L.A., Smith, K.A., Filbey, K.J., Harcus, Y., Hewitson, J.P., Redpath, S.A., Valdez, Y., Yebra, M.J., Finlay, B.B., and Maizels, R.M. (2014). Commensal-pathogen interactions in the intestinal tract: lactobacilli promote infection with, and are promoted by, helminth parasites. Gut Microbes *5*, 522–532.

Richmond, B.L., Boileau, A.C., Zheng, S., Huggins, K.W., Granholm, N.A., Tso, P., and Hui, D.Y. (2001). Compensatory phospholipid digestion is required for cholesterol absorption in pancreatic phospholipase A(2)-deficient mice. Gastroenterology *120*, 1193–1202.

Sato, T., Vries, R.G., Snippert, H.J., van de Wetering, M., Barker, N., Stange, D.E., van Es, J.H., Abo, A., Kujala, P., Peters, P.J., and Clevers, H. (2009). Single Lgr5 stem cells build crypt-villus structures in vitro without a mesenchymal niche. Nature *459*, 262–265.

Sommerville, R.I., and Bailey, M.A. (1973). Nematospiroides dubius: exsheathment of infective juveniles. Exp. Parasitol. *33*, 1–9.

Urban, J.F., Jr., Katona, I.M., and Finkelman, F.D. (1991a). Heligmosomoides polygyrus: CD4+ but not CD8+ T cells regulate the IgE response and protective immunity in mice. Exp. Parasitol. *73*, 500–511.

Urban, J.F., Jr., Katona, I.M., Paul, W.E., and Finkelman, F.D. (1991b). Interleukin 4 is important in protective immunity to a gastrointestinal nematode infection in mice. Proc. Natl. Acad. Sci. USA *88*, 5513–5517.

Valanparambil, R.M., Segura, M., Tam, M., Jardim, A., Geary, T.G., and Stevenson, M.M. (2014). Production and analysis of immunomodulatory excretory-secretory products from the mouse gastrointestinal nematode Heligmosomoides polygyrus bakeri. Nat. Protoc. *9*, 2740–2754.

von Moltke, J., Ji, M., Liang, H.E., and Locksley, R.M. (2016). Tuft-cell-derived IL-25 regulates an intestinal ILC2-epithelial response circuit. Nature *529*, 221–225.

Wang, S., Zhang, S., Liou, L.C., Ren, Q., Zhang, Z., Caldwell, G.A., Caldwell, K.A., and Witt, S.N. (2014). Phosphatidylethanolamine deficiency disrupts α-synuclein homeostasis in yeast and worm models of Parkinson disease. Proc. Natl. Acad. Sci. USA *111*, E3976–E3985.

Weinrauch, Y., Abad, C., Liang, N.S., Lowry, S.F., and Weiss, J. (1998). Mobilization of potent plasma bactericidal activity during systemic bacterial challenge. Role of group IIA phospholipase A2. J. Clin. Invest. *102*, 633–638.

Wellner, N., Diep, T.A., Janfelt, C., and Hansen, H.S. (2013). N-acylation of phosphatidylethanolamine and its biological functions in mammals. Biochim. Biophys. Acta *1831*, 652–662.

Wilson, M.S., Taylor, M.D., Balic, A., Finney, C.A., Lamb, J.R., and Maizels, R.M. (2005). Suppression of allergic airway inflammation by helminth-induced regulatory T cells. J. Exp. Med. *202*, 1199–1212.

Zaiss, M.M., Rapin, A., Lebon, L., Dubey, L.K., Mosconi, I., Sarter, K., Piersigilli, A., Menin, L., Walker, A.W., Rougemont, J., et al. (2015). The intestinal microbiota contributes to the ability of helminths to modulate allergic inflammation. Immunity *43*, 998–1010.

STAR★METHODS

KEY RESOURCES TABLE

REAGENT or RESOURCE	SOURCE	IDENTIFIER
Antibodies		
In Vivo*Mab* anti-mouse IL-4 (Clone 11B11)	BioXCell	Cat# BE0045; RRID: AB_1107707
In Vivo*Mab* Rat IgG1 (Clone HPRN)	BioXCell	Cat# BE0088; RRID: AB_1107775
Chemicals, Peptides, and Recombinant Proteins		
Recombinant mouse PLA2g1B protein	Elabscience	Cat# PKSM040953
Gentamicin Sulfate	Sigma	Cat# G3632
Metronidazole	Sigma	Cat# M3761
Cefloxin Sodium Salt	Santa Cruz Biotechnology	Cat# sc-217858
Vancomycin Hydrochloride	Sigma	Cat# SBR00001
Manoalide	Santa Cruz Biotechnology	Cat# sc-200733
Critical Commercial Assays		
Encore® Complete RNA-Seq Library Systems kit	NuGEN	Cat#0311, 0312, 0333, 0334.
EnzChek® Phospholipase A_2 Assay Kit	Invitrogen	Cat#E10217
CellTiter-Glo® Luminescent Cell Viability ATP Assay	Promega	Cat#G7571
Deposited Data		
Raw and analyzed RNaseq data	This paper	GEO: GSE102789 https://www.ncbi.nlm.nih.gov/geo/query/acc.cgi?acc=GSE102789
Metabolomics data	This paper	EMBL-EBI MetaboLights: MTBLS523 and PubMed PMID: 23109552 http://www.ebi.ac.uk/metabolights/MTBLS523
Experimental Models: Organisms/Strains		
Mouse: *Pla2g1b*$^{-/-}$ (C57BL/6 background)	David Hui, Richmond et al., 2001	N/A
Mouse: *Pla2g1b*$^{-/-}$*Il4*^{gfp} (C57BL/6 background)	This Paper	N/A
Mouse: C57BL/6	The Francis Crick Institute	N/A
Mouse: *Rag*$^{-/-}$*cg*$^{-/-}$(C57BL/6 background)	The Francis Crick Institute	N/A
Mouse: *Rag2*$^{-/-}$ (C57BL/6 background)	The Francis Crick Institute	N/A
Heligmosomoides polygyrus bakeri	The Francis Crick Institute	N/A
Nippostrongylus brasiliensis	London School of Hygiene and Tropical Medicine	N/A
Trichuris muris	The Francis Crick Institute	N/A
Oligonucleotides		
Hprt Primers, see Table S4.	This paper	N/A
Pla2g1b Primers, see Table S4.	This paper	N/A
Arg1 Primers, see Table S4.	This paper	N/A
Retnla Primers, see Table S4.	This paper	N/A
Chi3l3 Primers, see Table S4.	This paper	N/A
Retnlb Primers, see Table S4.	This paper	N/A
Gob5 Primers, see Table S4.	This paper	N/A
Software and Algorithms		
FastQC	Babraham Bioinformatics	www.bioinformatics.babraham.ac.uk/projects/fastqc
Trimmomatic	N/A	http://www.usadellab.org/cms/index.php?page=trimmomatic
Tophat2	N/A	http://ccb.jhu.edu/software/tophat

(*Continued on next page*)

Continued

REAGENT or RESOURCE	SOURCE	IDENTIFIER
Ingenuity Pathway Analysis® (IPA®)	QIAGEN	N/A
Xcalibur 3.0.63	Thermo Fisher-Scientific	N/A
Progenesis	Nonlinear Dynamics	N/A
CEU Mass Mediator	CEMBIO, Universidad San Pablo	http://ceumass.eps.uspceu.es/mediator/
Other		
mm10 (Ensembl version 75)	Ensembl	N/A

CONTACT FOR REAGENT AND RESOURCE SHARING

Further information and requests for resources and reagents should be directed to and will be fulfilled by the Lead Contact, Mark Wilson (wilson.mark@gene.com)

EXPERIMENTAL MODEL AND SUBJECT DETAILS

Animal Strains

All mice used in this study were maintained under specific pathogen-free conditions at the Mill Hill Laboratory, The Francis Crick Institute (London, UK). C57BL/6, *Pla2g1b*$^{-/-}$ (Richmond et al., 2001), 4get (Mohrs et al., 2001), *Rag*$^{-/-}$*cg*$^{-/-}$ and *Rag2*$^{-/-}$ mice were bred and maintained at The Francis Crick Institute. *Pla2g1b*$^{-/-}$*Il4*^{gfp} mice were generated by crossing *Pla2g1b*$^{-/-}$ and 4get mice at The Francis Crick Institute. All mice used were male and between 6-12 weeks old at the start of the experiment and were not involved in any previous procedures. Animal experiments were performed according to institutional guidelines and following UK Home Office regulations (project license 70/8809) and were approved by The Francis Crick Institute Ethical Review Panel.

Organoid Culture

Mouse organoids were established and maintained at 37°C as three-dimensional spheroid culture in Matrigel (R&D system) from isolated crypts collected from the duodenum of male C57BL/6 mice. The basic culture medium (ENR) contained advanced DMEM/F12 supplemented with penicillin/streptomycin, 10 mM HEPES, 2mM Glutamax, B27 (all from Life Technologies) and 1cmM N-acetylcysteine (Sigma) supplemented with murine recombinant EGF (life technologies), R-spondin1-CM (Trevigen) (10% final volume) and Noggin-CM (kindly provided by Dr. Hans Clevers, Hubrecht Institute, Utrecht, the Netherlands) (20% v/v). Wnt3a-CM was used at 50% (v/v) for 7 days at the beginning of the culture, then withdrawn. Organoids were stimulated with 20 ng/mL IL-4 (R&D) and 20ng/mL IL-13 (R&D) for 48 hr. RNA was extracted and qRT-PCR performed as described below.

Bone Marrow-derived Macrophage (BMDM) Culture and Stimulation

Bone marrow was isolated from the femur and tibia of mice and the red blood cells were lysed with ACK lysis buffer (GIBCO). The remaining cells were then cultured in DMEM (GIBCO) (with 20% L929 cell media (in-house preparation), 10% FCS (Invitrogen), 1% L-Glutamine (GIBCO), 100 U/mL Penicillin and 100 μg/mL Streptomycin (GIBCO), 10 mM HEPES (Lonza) and 0.05mM 2-mercaptoethanol (GIBCO)) in 10mls at a density of 5x10^5 cells/ml at 37°C. After 7 days of culture non-adhesive cells were removed before removing adherent BMDMs using 2.5mM edta (Invitrogen) in PBS (GIBCO) with 5% FCS (Invitrogen). Adherent BMDMS were washed and resuspended in DMEM (with 1% FCS (Invitrogen), 1% L-Glutamine (GIBCO), 100 U/mL Penicillin and 100 μg/mL Streptomycin (GIBCO), 10 μM HEPES (Lonza) and 0.05mM 2-mercaptoethanol (GIBCO)). Adherent BMDMs were then plated at a density of 2x10^6 cells/ml for 24 hr. The adherent BMDMs were then stimulated for 24 hr with either 20ng/ml IL-4 (R&D) and 20ng/ml IL-13 (R&D) before RNA extraction.

METHOD DETAILS

Parasite Infections and PLA$_2$g1B Treatment

Heligmosomoides polygyrus bakeri

Mice were infected with 200 L3 infective *H. polygyrus* larvae *(p.o.)* on day 0 (1° infection). Mice were drug cured (Rx) with the anthelminthic drug Pyrantel Embonate (2.5 mg/dose, Pfizer) *(p.o.)* on days 14 and 15. Mice were secondary (2°) challenge infected on day 35 or day 56 with 200 L3 infective *H. polygyrus* larvae *(p.o.)*. *H. polygyrus* worms were counted in the wall of the intestine at day 5 post infection and luminal worms were counted 14 days-post 1° or 2° infection.

Nippostrongylus brasiliensis

Mice were infected with 350 L3 infective *N. brasiliensis larvae* (*s.c.*) on day 0. Luminal *N. brasiliensis* worms were counted on day 8.

Trichuris muris

Mice were infected with 200 embryonated *T. muris* eggs (*p.o.*) on day 0. Luminal *T. muris* worms were counted on day 35. For PLA_2g1B treatment, infective L3 *H. polygyrus* larvae were exsheathed as previously described (Sommerville and Bailey, 1973). Briefly, 0.85% w/v NaCl (in distilled water) was bubbled with 40% CO_2 in Nitrogen for 5 min, the tube was then sealed and incubated in a 37°C waterbath for a further 5 min. The pH was adjusted to pH2 using HCl and 5 mL was added to 30000-50000 L3 larvae before bubbling with 40% CO_2 in Nitrogen for 30 s. The tube was then sealed and incubated in a 37°C waterbath for a further 30 min. Exsheathed L3 larvae were washed and counted before treatment with PLA_2g1B. 8000 exsheathed L3 *H. polygyrus* larvae were treated with recombinant mouse PLA_2g1B (Elabscience), recombinant mouse PLA_2g1B plus manoalide (200 ng/μL final concentration, Santa Cruz Biotechnology) or heat inactivated recombinant mouse PLA_2g1B in 1mL EnzChek® PLA_2 reaction buffer (Invitrogen) at room temperature for 24 hr. Recombinant mouse PLA_2g1B was heat inactivated by heating at 100°C for 4 hr. Following treatment, larvae were washed with MilliQ water prior to use in ATP assay (detailed below) or infection. Adult L5 *H. polygyrus* worms were isolated from C57BL/6 following primary infection between days 14 and 28 using a modified Baermann apparatus. Adult L5 *H. polygyrus* worms were treated with recombinant mouse PLA_2g1B (Creative Biomart) or heat inactivated recombinant mouse PLA_2g1B in EnzChek® PLA_2 reaction buffer (Invitrogen) at room temperature for 24 hr.

ATP Assay

The ATP of infective L3 *H. polygyrus* larvae, L4 *H. polygyrus* larvae (removed from intestinal wall at day 7 post infection) and adult L5 *H. polygyrus* worms was measured using the CellTiter-Glo® Luminescent Cell Viability Assay (Promega). Briefly, *H. polygyrus* adult worms, two L4 larvae or 100 L3 larvae were homogenized using a motorised pestle in 110 μL of PBS and 110 μL of CellTiter-Glo® Reagent. The homogenate was incubated for 10 min at room temperature before centrifugation at 1000 g for 3 min. 200 μL of the supernatant was transferred to a 96 well opaque-walled plate and incubated for 10 min at room temperature before recording luminescence. An ATP standard curve was generated by using recombinant ATP (Promega) as detailed in the CellTiter-Glo® Luminescent Cell Viability Assay instructions.

Antibiotic Treatment

The antibiotics Gentamicin sulfate salt (1 mg/mL, Sigma), Metronidazole (1 mg/mL, Sigma), Cefloxin sodium salt (1 mg/mL, Santa Cruz Biotechnology), Vancomycin hydrochloride (1 mg/mL, Sigma) were administered in the drinking water. Treatment was started 7 days prior to 1° *H. polygyrus* infection and maintained throughout the duration of the experiment.

Antibody Treatment

Anti-IL-4 antibody (0.5 mg/dose, BioXcell) was administered *i.p.* on days 13, 15, 17, 19 and 21 after 1° *H.* polygyrus infection. Mice were drug cured (Rx) with the anthelminthic drug Pyrantel Embonate (2.5 mg/dose, Pfizer) *(p.o.)* on days 14 and 15.

Histology, In Situ Hybridization

Small intestinal tissue was removed and fixed in 4% formaldehyde for 24 hr then washed in 70% ethanol. The tissues were embedded in paraffin, and sectioned. Sections were stained with hematoxylin and eosin stain or Alcian blue/ periodic acid-Schiff stain. *Pla2g1b* Staining: *Pla2g1b* RNAscope® probes were designed by Advanced Cell Diagnostics and in situ hybridization was performed with RNAscope® 2.5 Reagent Kit Brown (Advanced Cell Diagnostics), following the manufacturer's instructions. The final *Pla2g1b* signal was detected chromogenically using DAB and the sections were counterstained with Haematoxylin Stained. Stained slides were scanned with a VS120-SL slide scanner (Olympus, Tokyo, Japan) and images were captured with the OlyVIA image viewer (Olympus).

RNA Sequencing and Analysis

RNA was extracted using the QIAGEN® miRNeasy Mini Kit, following the manufacturer's instructions. RNA integrity was confirmed using Agilent's 2100 Bioanalyser. Total RNA libraries were created using the Encore® Complete RNA-Seq Library Systems kit (NuGEN), following manufacturer's instructions. Total RNA libraries were sequenced using the Illumina® HiSeq 2500. The raw Illumina reads were analyzed as follows. First, the data quality was analyzed using FastQC (www.bioinformatics.babraham.ac.uk/projects/fastqc). Then the low quality bases were trimmed using Trimmomatic. The read pairs which passed the trimming quality filters were then aligned to mm10 (Ensembl version 75) using Tophat2. Counts were determined using htseq_count. Normalization and statistical analysis was performed using edgeR. Differential gene analysis was calculated from naive control group. Statistically significant genes with FDR < 0.05 are reported. Ingenuity Pathway Analysis® (IPA®): RNA sequencing datasets were uploaded to IPA® where fold change filters and pathway analysis algorithms were applied. Ratio of ratios plots were generated from the ratio of expression of genes from *H.p.* 2° to *H.p.* 1° (relative to naive, 2-fold filter, $p < 0.05$) (y axis) against the fold-change of each gene in both compared to naive (2-fold filter, $p < 0.05$) (x axis).

Quantitative Real-time Polymerase Chain Reaction

RNA was extracted and purified from tissue or cells as described above. Reverse transcription was performed with 0.1-1 μg RNA using QIAGEN® Quantitect RT Kit following manufacturer's instructions to create cDNA. Generated cDNA was used for quantitative real-time PCR analysis using Power SYBR® Green PCR Master Mix (Applied Biosystems) and quantified on the 7900HT (Applied

Biosystems). Where appropriate, relative gene expression was determined via normalization to the housekeeping gene *Hprt* and the relevant control group (see Figure legends and Table S4).

PLA_2 Activity Assay

PLA_2 activity was determined using EnzChek® Phospholipase A_2 Assay Kit (Invitrogen), following manufactures instructions. Briefly, approximately 1cm of duodenal tissue was removed and homogenized in 300 μL of cOmplete protease inhibitor (Roche) before centrifugation. 25 μL of the supernatant was transferred to a 96 XXX well plate with 25 μL of the substrate-liposome mix then incubated at room temperature in the dark for 10 min. Fluorescence emission was measured at 515nm and reported after blank reduction.

Cell Isolation

The spleen, mLNs and thymus were made into single-cell suspensions by gently mashing through a 40 micron filter (Thermo-Scientific, Loughborough, UK), and the red blood cells were lysed from the spleen single cell suspension with ACK lysis buffer (GIBCO). Single cell suspensions were used for *ex* vivo restimulations and flow cytometry analysis. For the isolation of small intestinal epithelial cells: adipose tissue was removed from the small intestine dissected longitudinally to remove faecal contents, and cut into 2cm segments. The epithelial layer was then dissociated by in incubating the intestine segments in PBS containing 10% FBS, 15cmm HEPES, 5cmm EDTA (Life Technologies, Paisley, UK), and 1cmm dithiothreitol (Sigma, Gillingham, UK) for 30cminutes at 37c°C. The remaining intestinal tissue was removed using a wide mesh sieve and epithelial layer was retained. Cells were layered onto 20% isotonic Percoll (GE Healthcare, Little Chalfont, UK) to remove debris. Cells were then resuspended in cIMDM (complete Iscove's Modified Dulbecco's Medium (cIMDM) containing 1% fetal bovine serum (FBS), 1mM EDTA, 100U/ml Penicillin (GIBCO) and 100 μg/ml Streptomycin (GIBCO), 8mM L-glutamine (GIBCO) and 0.05mM 2-mercaptoethanol (GIBCO)) and prepared for cell sorting.

Flow Cytometry and Cell Sorting

Cell sorting was performed using a MoFlo XDP cell sorter (Beckman Coulter). Cell suspensions were stained for 25 min with antibodies in PBS with 1% FCS. To prepare for sorting, stained cells were diluted in phenol-red free IMDM (GIBCO) (with 1% FCS, 2mM EDTA (Invitrogen), 100 U/mL Penicillin and 100 μg/mL Streptomycin (GIBCO), 8 mM L-glutamine (GIBCO), and 0.05 mM 2-mercaptoethanol (GIBCO)). For flow cytometry analysis, cells were analyzed using a BD LSRFortessa X-20 (BD Biosciences) or BD LSRII (BD Biosciences) and data were analyzed using FlowJo software (Version 10, Treestar Inc). Cells were sometimes fixed in 2%–4% paraformaldehyde for FACS analysis. For cell sorting, viability of the cells was determined using Propidium Iodide (Sigma); for analysis, viability of the cells was determined using the LIVE/DEAD Fixable Blue kit (Life Technologies). Antibodies used include: CD3 (145-2C11; APC, (BioLegend)), CD4 (RM4-5; BV605, efluor450 (eBioscience), APC (BioLegend)), CD8 (53-6.7; PE-Cy7, APC (BioLegend)), CD11c (N418; APC (BioLegend)), CD11b (M1/70; APC (BioLegend)), CD19 (1D3; efluor450 (eBioscience)), CD19 (6D5; APC (BioLegend)), CD25 (PC61; APC-Cy7 (BioLegend), APC (eBioscience)), CD44 (IM7; Percpcy5.5 (eBioscience)), CD45 (30-F11; FITC (Bioscience)), CD49b (DX5; APC (BioLegend)), CD62L (MEL-14; APC (eBioscience)), CD69 (H1.2F3; PE (BioLegend)), EpCam (G8.8; APC (eBioscience), Foxp3 (FJK-16S; PE (eBioscience)), Gr1 (RB68C5; APC (BioLegend)), IFNγ (XMG1.2; PE (BD Bioscience)), IL4 (PE, 11B11, eBioscience)), IL17a (17B7; PE-Cy7 (eBioscience)), KLRG1 (2F1; PerCpefluor710 (eBioscience)), NK1.1 (PK136; APC (BioLegend)), Sca1 (E13-161.7; PB (eBioscience)), TCR$\gamma\delta$ (GL3; APC (BioLegend)), TCR$\alpha\beta$ (H57-597; APC (eBioscience)), TCRβ (H57-597; Percpcy5.5 (BioLegend)), Ter119 (TER-119; APC (BioLegend)), Thy1.2 (53-2.1; PE-Cy7 (BioLegend)). All staining was performed in the presence of FcR Blocking Reagent (Miltenyi Biotec). Intracellular cytokine staining (ICS) was performed following 6 hr of re-stimulation with 50ng/mL phorbol 12-myristate 13-acetate (PMA, Promega) and 1 μg/mL ionomycin (Sigma) and BD Golgi Stop and BD Golgi Plug (diluted 1:1000, BD Biosciences). Following surface stain, cells were incubated with eBioscience Fixation/Permeabilization buffer for 25 min followed by 25 min in Permeabilization buffer (eBioscience), and incubation with antibodies in Permeabilization buffer for a further 30 min. ILCs were analyzed using the following strategy: Live, lymphocytes, $CD45^+$, $Lineage^-$ (CD3, CD4, CD8, CD19, CD11c, CD11b, NK1.1, TCRβ, TCR$\gamma\delta$, Gr-1, CD49b, Ter119), $Thy1.2^+$, $KLRG1^+$, and $Sca1^+$.

Tregs were analyzed using the following gating strategy: Live, lymphocytes, $CD4^+$, $TCR\beta^+$, $CD25^+$ and $Foxp3^+$. Epithelial cells were sorted using the following gating strategy: Live, $CD45^-$ and $EpCam^+$.

Ex Vivo Stimulations

mLNs were harvested and processed as above. Cells were plated at $2x10^5$ cells per 200μl with 10μg/ml of *H. polygyrus* antigen extract (HEX). Supernatant was harvested after 4 days. Cytokines were detected in the supernatant using ELISAs.

ELISAs

IFNγ, IL-5 and IL-13 were measured using DuoSet ELISA kits, according to the manufacturer's instructions (R&D). Cysteinyl leukotrienes and prostaglandin E_2 were measured in small intestinal homogenate (see above) using ELISA kits, according to manufacturer's instructions (Enzo), and normalized to total protein content. Total IgE ELISA was performed by coating with Purified Rat Anti-Mouse IgE (R35-72, BD PharMingen) at 2 μg/mL overnight, followed by overnight incubation with serum and standard (Purified Mouse IgE,k isotype Standard, BD PharMingen), and detection with Biotin Rat Anti-Mouse IgE at 1 μg/mL (R35-118, BD PharMingen), Streptavidin HRP at 1:000 (BD PharMingen) and ABTS One Component HRP Microwell Substrate (SurModics). *H. polygyrus*-specific IgG1 was detected by coating plates with 5 μg/mL *H. polygyrus* antigen overnight, followed by

overnight incubation with serially diluted serum and detection with Biotin Rat Anti-Mouse IgG1 (Invitrogen) and streptavidin and ABTS, as above.

Scanning Electron Microscopy (SEM)

H. polygyrus larvae were dehydrated stepwise in ethanol (2 × 5 min in 70, 90 and 100% ethanol) before transferring to 100% acetone (2 × 5 min). Larvae were critical point dried from 100% Ethanol in a CPD300 critical point drier (Leica Microsystems UK), mounted on a carbon sticky pad on a stub, sputter-coated with 5 nm of platinum, and imaged in a Phenom ProX benchtop scanning electron microscope (Phenom-World) with a secondary electron detector.

Serum Chemistry

Whole blood was collected from mice and the serum separated after clotting. The serum was sent to the MRC Harwell Institute (UK) for metabolite analysis. Lysophosphatidylcholine (LPC) was measured using the AZWELL LPC Assay Kit according to the manufacturer's instructions (Cosmo Bio).

Lipid Extraction and Analysis

Lipids were extracted from *H. polygyrus* L3 larvae using a method adapted from a previous publication (Meyer et al., 1966). Following PLA_2g1B treatment ~8000 *H. polygyrus* larvae were washed three times with MilliQ water, resuspended in 2 mL methanol and heated in a sealed tube under nitrogen at 55°C for 20 min. After cooling, 4 mL chloroform was added and the sample was agitated with a magnetic stirrer for 3 hr. The organic phase was removed and the residue ground (using a glass homogenizer) and extracted with 2 mL chloroform/methanol (2:1, v/v) for 2 hr.

For LC-MS, lipids were dried under nitrogen and redissolved in 100 μL of methanol/chloroform (1:1 v/v) and diluted 1:2 with solvent A (hexane:isopropanol, 70:30 [v: v], 0.02% [m/v] formic acid, 0.01% [m/v] ammonium hydroxide), centrifuged at 1,500 rpm for 5 min to remove trace non-lipidic materials prior to transfer to a glass autosampler vial (Agilent). 10 μL was injected onto a BETASIL diol column (5 um x 150 mm x 2.1 mm, with BETASIL diol guard column (10 mm x 2.1 mm), held at 20°C) in an Ultimate 3000 HPLC system coupled to a Thermo Exactive Plus Orbitrap MSfor full scan or Q Exactive Orbitrap MS for MS/MS scan. Lipids were eluted at 0.15 ml/min with a binary gradient from 0% to 100% solvent B (isopropanol:methanol, 70:30 [v/v], 0.02% [m/v] formic acid, 0.01% [m/v] ammonium hydroxide): 0–10 min, 0% B; 17–22 min, 50% B; 30–35 min, 100% B; 40–44 min, 0% B, followed by additional 6 min 0% B post-run. MS data were acquired in both polarities using a full scan method. The positive and negative HESI-II spray voltages were 4.5 and 3.5 kV, respectively; the heated capillary temperature was 250°C; the sheath gas pressure was 30 psi; the auxiliary gas setting was 20 psi; and the heated vaporizer temperature was 150°C. Both the sheath gas and the auxiliary gas were nitrogen. The parameters of the full mass scan were as follows: a resolution of 70,000, an auto gain control target under 3×10^6, a maximum isolation time of 200 ms, and an *m/z* range 200–3000. To confirm the identification of significant features, samples were re-ran in parallel reaction monitoring (PRM_ mode, parameters as follows: a resolution of 17,500, an auto gain control target under 2×10^5, a maximum isolation time of 100 ms, an isolation window of *m/z* 0.4 and normalized collision energy were optimized for each feature individually. Data were acquired using Xcalibur 3.0.63 (Thermo Fisher Scientific) and Progenesis (Nonlinear Dynamics) was used for data alignment and peak detection. Data were normalized against the total ion abundance.

Annotations were assigned to accurate masses with a maximum error of 5 ppm using Metlin, LipidMaps, Kegg and HMDB which were searched simultaneously using the CEU Mass Mediator engine (http://ceumass.eps.uspceu.es/mediator/).

Experimental Design

All experiments contained at least three biological replicates and are representative of at least two independent experiments (see figure legends for exact values). No strategy was employed for randomization, sample size estimation or data inclusion/exclusion criteria. The studies performed were also not blinded at any stage.

QUANTIFICATION AND STATISTICAL ANALYSIS

All statistical analysis for biological data was performed using GraphPad Prism (v6.02). Data was analyzed, where appropriate, with either an unpaired two-tailed t test, One-way ANOVA (Dunnett's multiple comparison analysis), Two-way ANOVA (Sidak's multiple comparison analysis) or Mann-Whitney test. n represents the number of biological replicates. Please see figure legends for statistical tests used and exact value of n. No methods were used to confirm whether the data met assumptions of the statistical approach used. Values are reported as the means ± SEM. * = $p < 0.05$, ** = $p < 0.01$, *** = $p < 0.001$ and **** = $p < 0.0001$.

DATA AND SOFTWARE AVAILABILITY

The raw and analyzed RNA sequencing data files have been deposited in the NCBI Gene Expression Omnibus database under ID code GEO: GSE102789. https://www.ncbi.nlm.nih.gov/geo/query/acc.cgi?acc=GSE102789

LC-MS metabolomics data have been deposited in the EMBL-EBI MetaboLights database under ID code MTBLS523 and PubMed PMID: 23109552. http://www.ebi.ac.uk/metabolights/MTBLS523

Cell Host & Microbe, Volume *22*

Supplemental Information

Epithelial-Cell-Derived Phospholipase A_2 Group 1B Is an Endogenous Anthelmintic

Lewis J. Entwistle, Victoria S. Pelly, Stephanie M. Coomes, Yashaswini Kannan, Jimena Perez-Lloret, Stephanie Czieso, Mariana Silva dos Santos, James I. MacRae, Lucy Collinson, Abdul Sesay, Nikolay Nikolov, Amina Metidji, Helena Helmby, David Y. Hui, and Mark S. Wilson

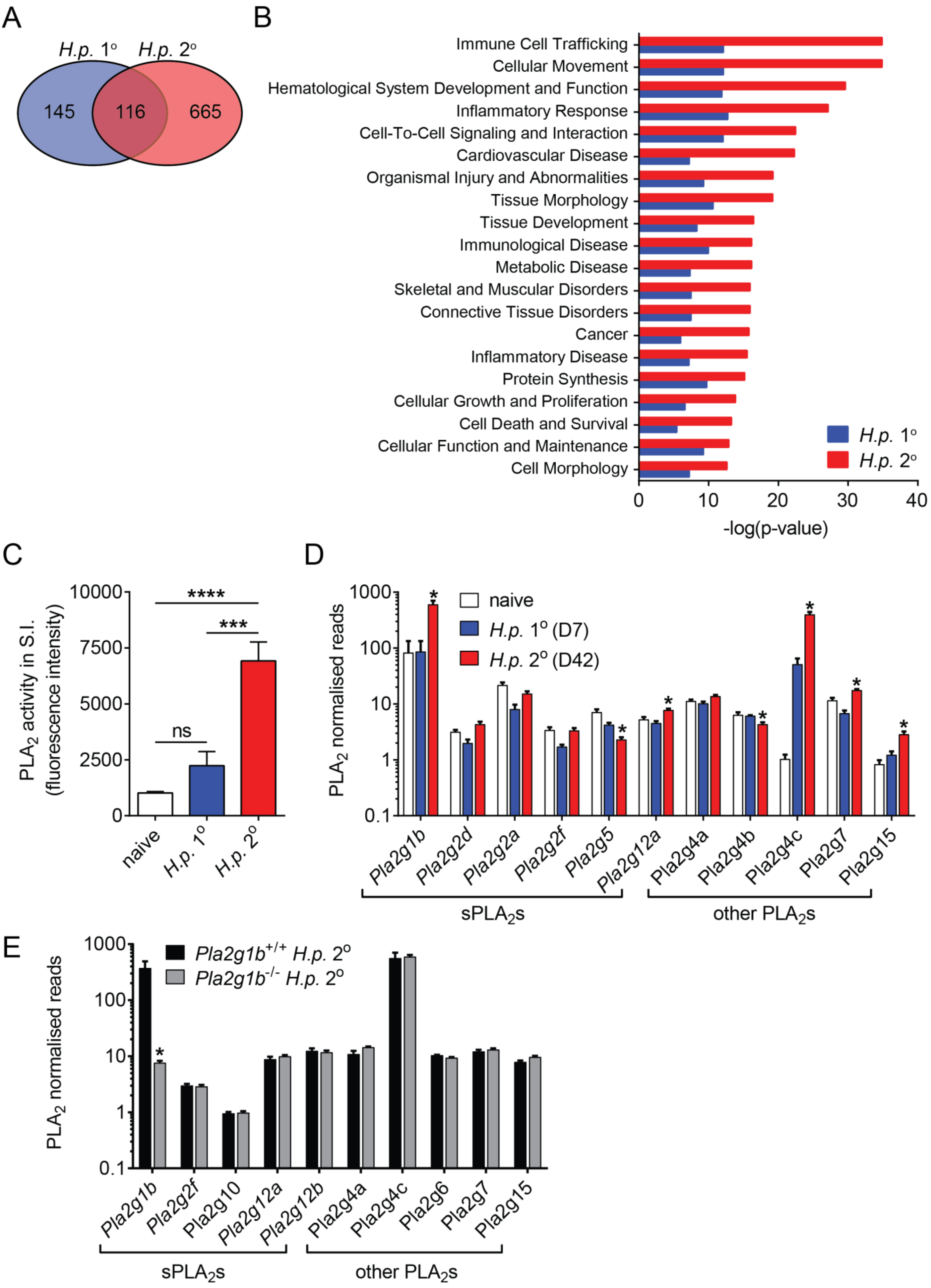
A
H.p. 1°
H.p. 2°
145
116
665
B
Immune Cell Trafficking
Cellular Movement
Hematological System Development and Function
Inflammatory Response
Cell-To-Cell Signaling and Interaction
Cardiovascular Disease
Organismal Injury and Abnormalities
Tissue Morphology
Tissue Development
Immunological Disease
Metabolic Disease
Skeletal and Muscular Disorders
Connective Tissue Disorders
Cancer
Inflammatory Disease
Protein Synthesis
Cellular Growth and Proliferation
Cell Death and Survival
Cellular Function and Maintenance
Cell Morphology
H.p. 1°
H.p. 2°
0
10
20
30
40
-log(p-value)
C
PLA2 activity in S.I. (fluorescence intensity)
10000
7500
5000
2500
0
****
***
ns
naive
H.p. 1°
H.p. 2°
D
PLA2 normalised reads
1000
100
10
1
0.1
naive
H.p. 1° (D7)
H.p. 2° (D42)
Pla2g1b
Pla2g2d
Pla2g2a
Pla2g2f
Pla2g5
Pla2g12a
Pla2g4a
Pla2g4b
Pla2g4c
Pla2g7
Pla2g15
sPLA2s
other PLA2s
E
PLA2 normalised reads
1000
100
10
1
0.1
Pla2g1b+/+ H.p. 2°
Pla2g1b-/- H.p. 2°
Pla2g1b
Pla2g2f
Pla2g10
Pla2g12a
Pla2g12b
Pla2g4a
Pla2g4c
Pla2g6
Pla2g7
Pla2g15
sPLA2s
other PLA2s

Figure S1. RNA sequencing analysis of 1º and 2º *H. polygyrus* infection. Related to Figure 1. (A) Common and differentially expressed genes between *H.p.* 1º and *H.p.* 2º (relative to naïve, 2-fold filter, $p<0.05$). (B) Top 20 pathways predicted to be activated in *H.p.* 2º (relative to strain naïve, 2-fold filter, $p<0.05$). (**C**) PLA_2 activity in the small intestine of naïve, *H.p.* 1º and *H.p.* 2º mice. (D & E) Phospholipase A_2 isoform expression in the small intestine from RNA sequencing. Data represented as mean ± SEM, n=6. ns = not significant, *** = $p<0.001$, **** = $p<0.0001$ determined using a one-way ANOVA with Tukey's multiple comparison analysis.

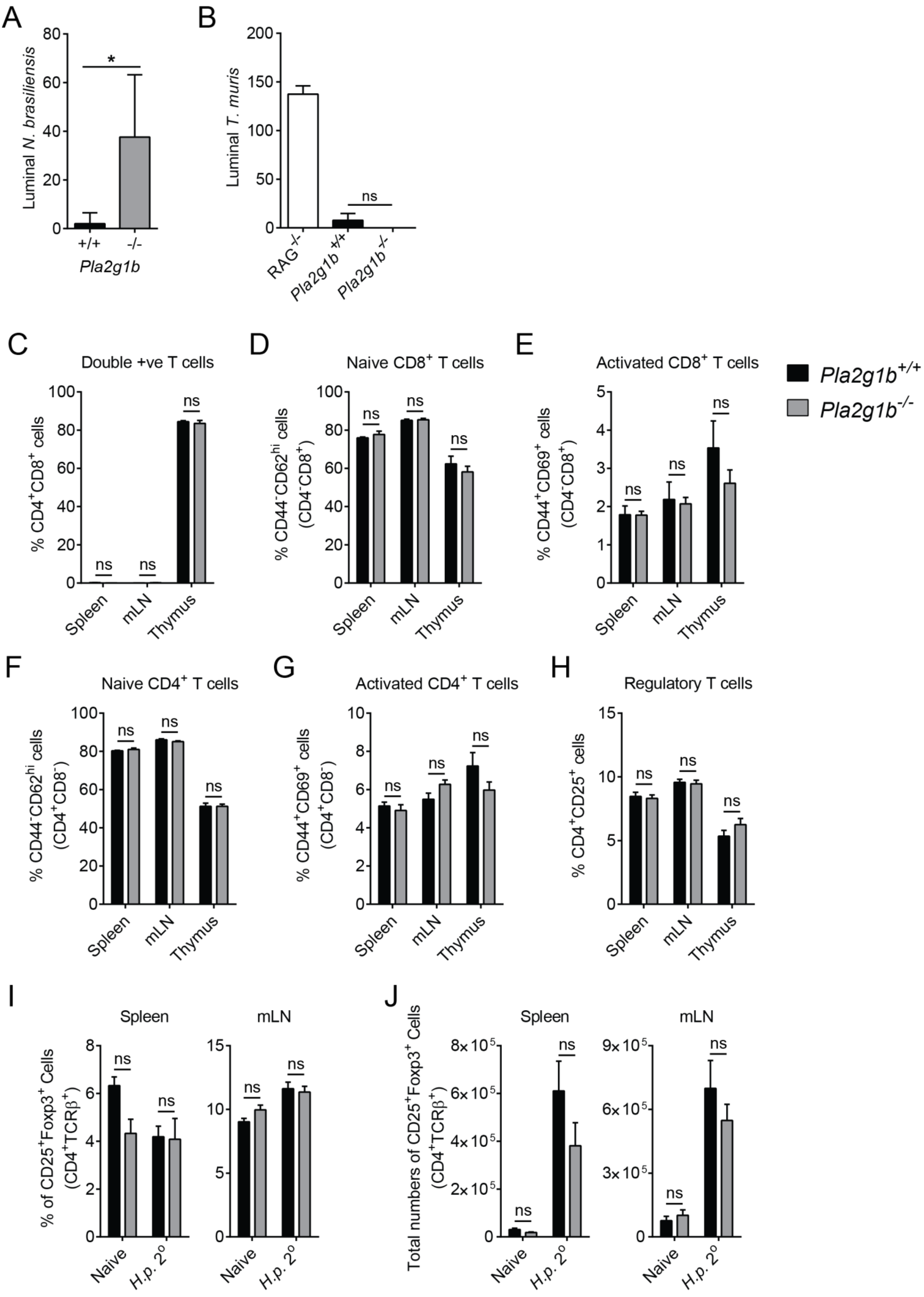
A
Luminal N. brasiliensis
+/+
-/-
Pla2g1b
B
Luminal T. muris
RAG-/-
Pla2g1b+/+
Pla2g1b-/-
ns
C
Double +ve T cells
% CD4+CD8+ cells
D
Naive CD8+ T cells
% CD44-CD62hi cells (CD4-CD8+)
E
Activated CD8+ T cells
% CD44+CD69+ cells (CD4-CD8+)
Pla2g1b+/+
Pla2g1b-/-
F
Naive CD4+ T cells
% CD44-CD62hi cells (CD4+CD8-)
G
Activated CD4+ T cells
% CD44+CD69+ cells (CD4+CD8-)
H
Regulatory T cells
% CD4+CD25+ cells
Spleen
mLN
Thymus
I
Spleen
mLN
% of CD25+Foxp3+ Cells (CD4+TCRβ+)
Naive
H.p. 2o
J
Spleen
mLN
Total numbers of CD25+Foxp3+ Cells (CD4+TCRβ+)
Naive
H.p. 2o

Figure S2. *Pla2g1b* is required for effective immunity to small intestine-dwelling helminths and T cells are intact in *Pla2g1b*$^{-/-}$ mice. Related to Figure 2.

(A) Luminal *N. brasiliensis* worms in the small intestine 8 days-post infection, n=5. (B) Luminal *T. muris* worms in the cecum and large intestine 35 days-post infection. *Rag*$^{-/-}$ mice were used as an additional control to confirm the infectious dose administered. T cell frequency was assessed in naïve mice (C-J). (C) $CD4^{+}CD8^{+}$ cells. (D) $CD44^{-}CD62^{hi}$ $CD8^{+}$ cells. (E) $CD44^{+}CD69^{+}$ $CD8^{+}$ cells. (F) $CD44^{-}CD62^{hi}$ $CD4^{+}$ cells. (G) $CD44^{+}CD69^{+}$ $CD4^{+}$ cells. (H) $CD4^{+}CD25^{+}$ cells. (I) Frequency and total number of $CD25^{+}Foxp3^{+}$ $CD4^{+}TCR\beta^{+}$ cells in the spleen and (J) mLN in naïve and 7 days-post 2° *H.* polygyrus infected mice. Data represented as mean ± SEM, n=5-6. All data is representative of at least two independent experiments. ns = not significant, * = p<0.05, determined using an unpaired two-tailed t test.

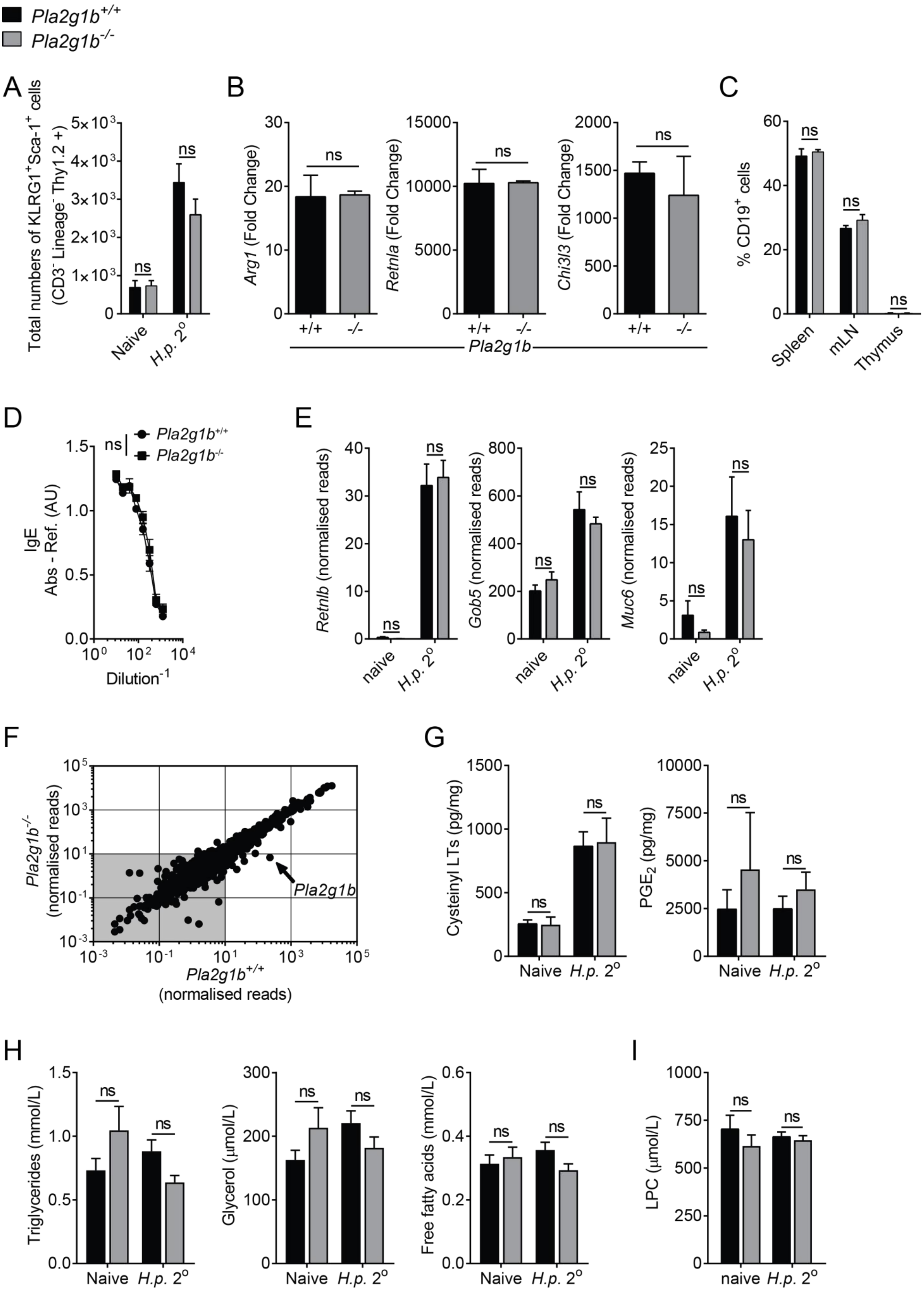
Pla2g1b+/+
Pla2g1b-/-
A
Total numbers of KLRG1+Sca-1+ cells (CD3- Lineage- Thy1.2 +)
Naive
H.p. 2o
ns
B
Arg1 (Fold Change)
Retnla (Fold Change)
Chi3l3 (Fold Change)
+/+
-/-
Pla2g1b
C
% CD19+ cells
Spleen
mLN
Thymus
D
IgE Abs - Ref. (AU)
Dilution-1
E
Retnlb (normalised reads)
Gob5 (normalised reads)
Muc6 (normalised reads)
naive
F
Pla2g1b-/- (normalised reads)
Pla2g1b+/+ (normalised reads)
Pla2g1b
G
Cysteinyl LTs (pg/mg)
PGE2 (pg/mg)
H
Triglycerides (mmol/L)
Glycerol (µmol/L)
Free fatty acids (mmol/L)
I
LPC (µmol/L)

Figure S3. Type 2 immunity, Bioactive lipids synthesis and lipid metabolism is intact in *Pla2g1b*$^{–/–}$ mice. Related to Figure 2.

(A) Total numbers of KLRG1^{+}Sca-1^{+}Lin^{-} cells in the mLN of naïve and 7 days-post 2º *H.* polygyrus infected mice. (B) Bone marrow-derived macrophages were stimulated with rIL-4 and rIL-13 for 24 hours and *Arg1*, *Retnla* and *Chi3l3* gene expression analysed. (**C**) Frequency of CD19^{+} cells were assessed in naïve mice. (D) IgE in the serum from mice 7 days-post 2º *H.* polygyrus infection. (E) Mucus-associated gene expression in the small intestine of WT and *Pla2g1b*$^{–/–}$ naïve and 7 days-post 2º *H.* polygyrus infected mice. (F) RNA sequencing-generated transcriptional landscape of the small intestine of naïve WT and *Pla2g1b*$^{–/–}$ mice (G) Cysteinyl leukotrienes (LTs) and Prostaglandin E_2 (PGE_2) concentration in the small intestine of naïve and 7 days-post 2º *H.* polygyrus infected mice. (H) Lipid metabolites in the serum of naïve and 7 days-post 2º *H.* polygyrus infected mice. (I) Lysophosphatidylcholine concentration in the serum of naïve and 7 days-post 2º *H.* polygyrus infected mice. Data represented as mean ± SEM, n=4-6. All data is representative of at least two independent experiments. ns = not significant, determined using a two-way ANOVA with Sidak's multiple comparison analysis or an unpaired two-tailed t test.

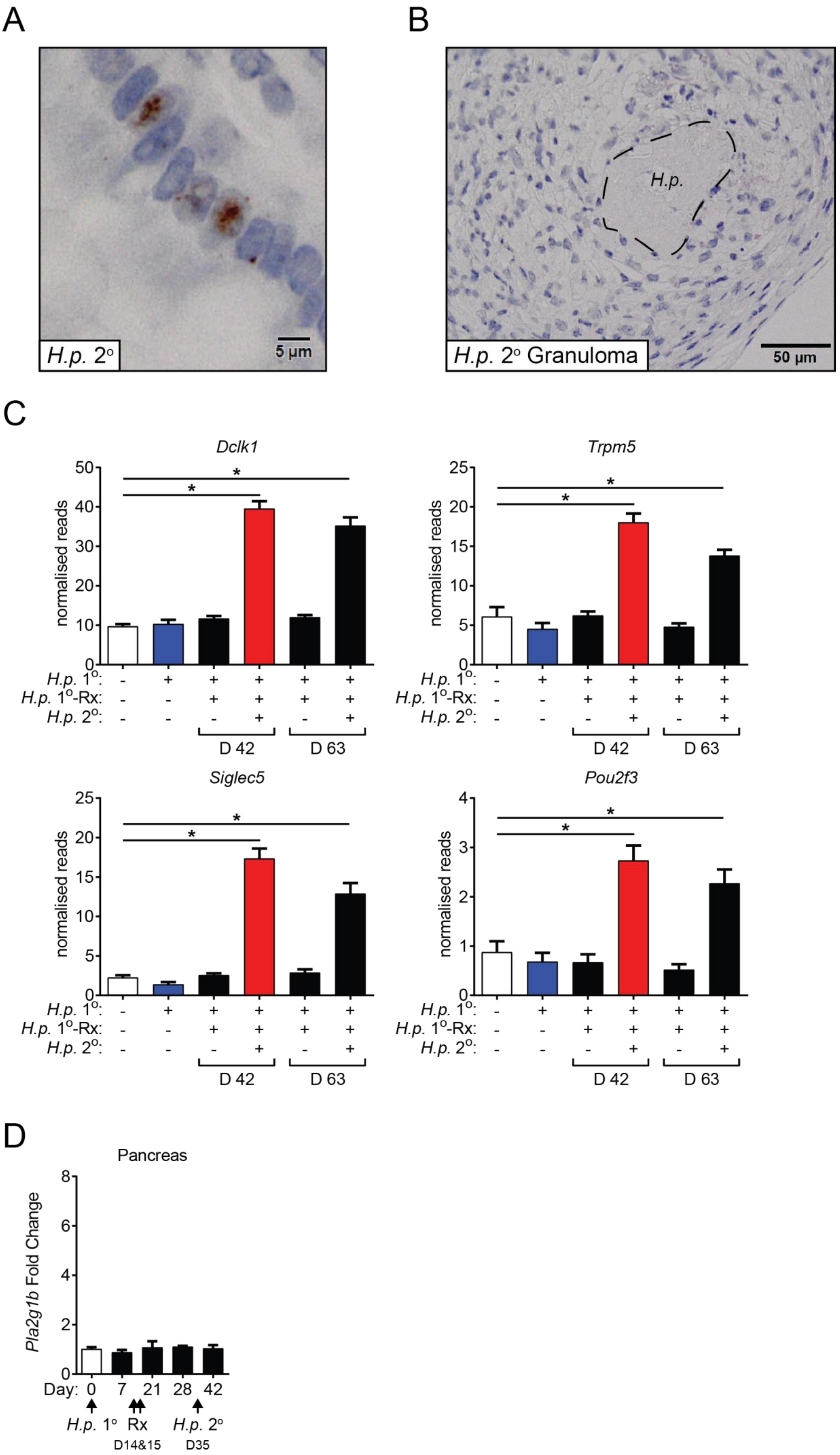
A
H.p. 2°
5 µm
B
H.p.
H.p. 2° Granuloma
50 µm
C
Dclk1
Trpm5
Siglec5
Pou2f3
normalised reads
H.p. 1°:
H.p. 1°-Rx:
H.p. 2°:
D 42
D 63
D
Pancreas
Pla2g1b Fold Change
Day: 0 7 21 28 42
H.p. 1°
Rx
D14&15
H.p. 2°
D35

Figure S4. *Pla2g1b* expression is restricted to epithelial cells in the small intestine of resistant mice. Related to Figure 4.

(A) *Pla2g1b* detection by RNAScope® ISH in the small intestine 7 days-post 2º *H.* polygyrus infection. (B) *Pla2g1b* detection by RNAScope® ISH in the granuloma surrounding *H. polygyrus* (*H.p.*) within the small intestine 7 days-post 2º *H.* polygyrus infection. (C) Tuft cell-specific gene expression data in small intestine from RNA sequencing data. (D) Kinetics of *Pla2g1b* expression in the pancreas during *H. polygyrus* 2º infection model, no significant difference detected using one-way ANOVA. Data represented as mean ± SEM, n=6-8. All data is representative of at least two independent experiments. * = $p<0.05$ determined within RNAseq analysis.

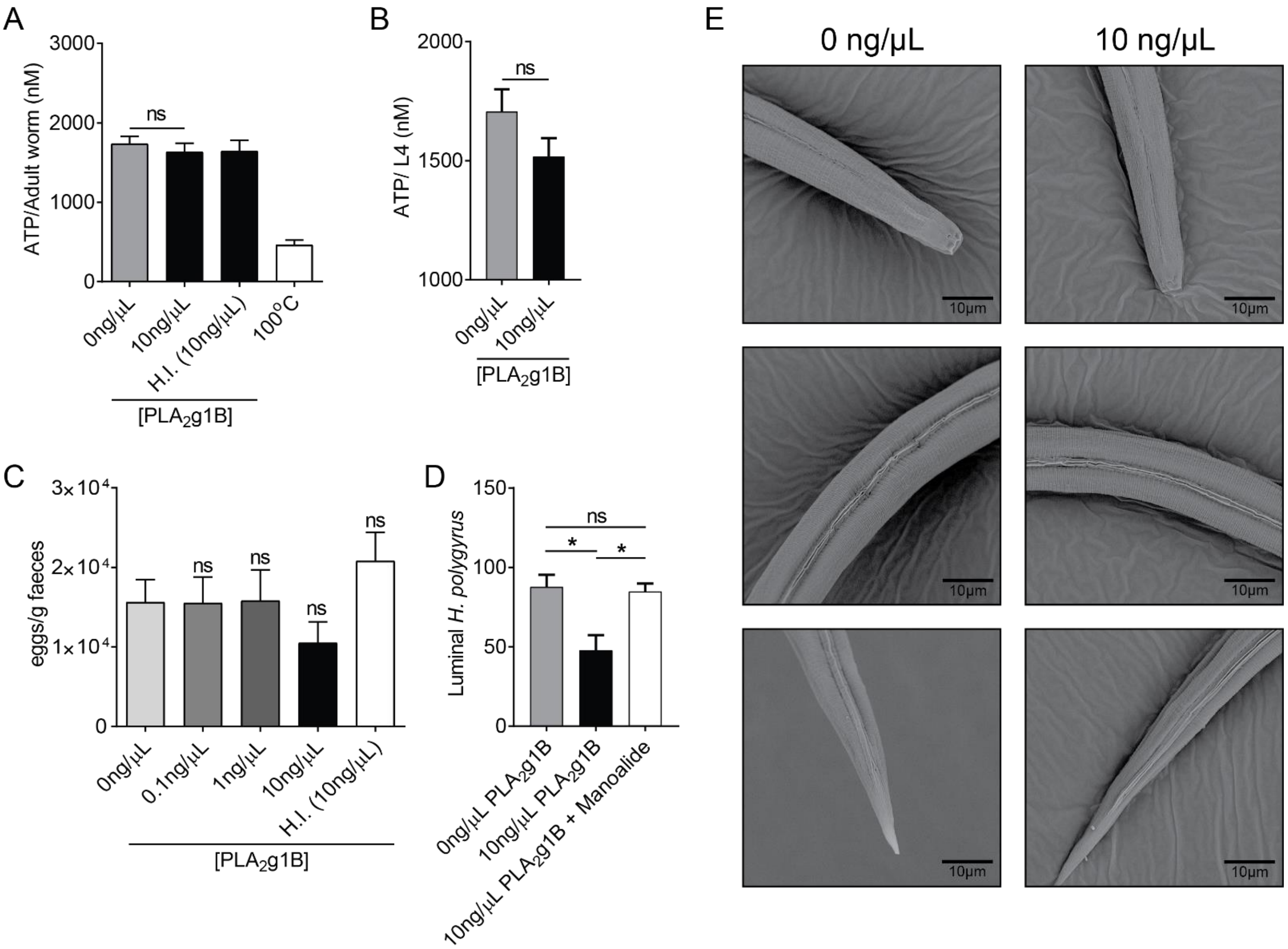

Figure S5. PLA$_2$g1B has direct anthelmintic properties against *H. polygyrus* L3 larvae. Related to Figure 3.

(A) ATP concentration of adult *H. polygyrus* worms following 24-hour treatment with rPLA$_2$g1B *in vitro*. (B) ATP concentration of L4 *H. polygyrus* isolated from the small intestine 14 days-post 1º infection following 24-hour treatment with rPLA$_2$g1B, n=40 (data pooled from three independent experiments). (C) Eggs recovered in the faeces 14 days-post 1º infection following 24-hour treatment with rPLA$_2$g1B, n=10 (data pooled from two independent experiments). (D) Luminal *H. polygyrus* worms in the small intestine 14 days-post 1º infection following 24-hour treatment with rPLA$_2$g1B ± Manoalide (200 ng ng/µL). (E) SEM of L3 *H.* polygyrus larvae after treatment with rPLA$_2$g1B. Data represented as mean ± SEM, n=5. All data is representative of at least two independent experiments. ns = not significant, * = $p<0.05$ determined using a one-way ANOVA with Tukey's or Dunnett's multiple comparison analysis or an unpaired two-tailed t-test.

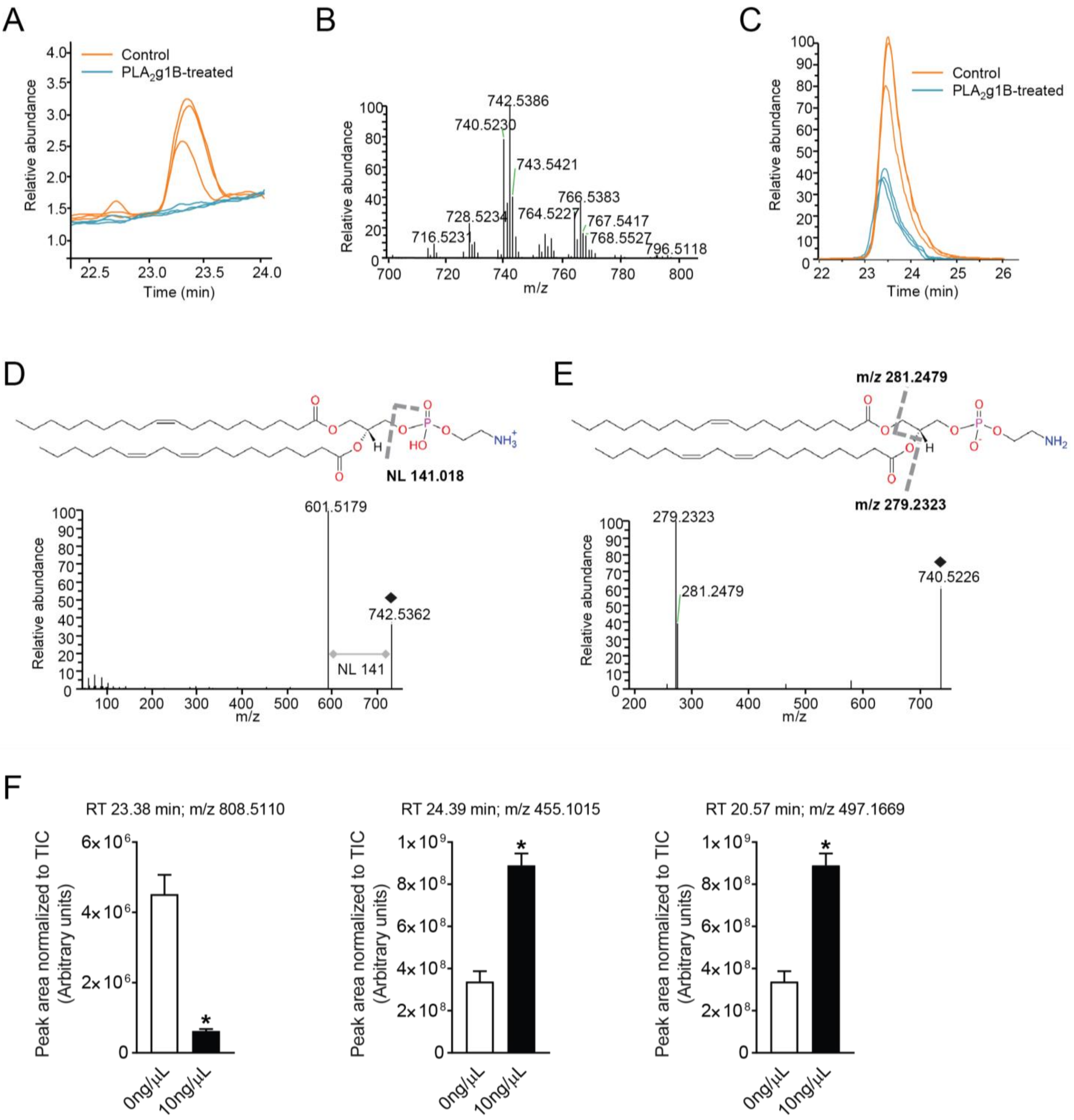

A
Control
PLA2g1B-treated
Relative abundance
Time (min)
B
Relative abundance
m/z
742.5386
740.5230
743.5421
728.5234
716.5231
766.5383
764.5227
767.5417
768.5527
796.5118
C
Control
PLA2g1B-treated
Relative abundance
Time (min)
D
NL 141.018
601.5179
742.5362
NL 141
Relative abundance
m/z
E
m/z 281.2479
m/z 279.2323
279.2323
281.2479
740.5226
Relative abundance
m/z
F
RT 23.38 min; m/z 808.5110
RT 24.39 min; m/z 455.1015
RT 20.57 min; m/z 497.1669
Peak area normalized to TIC (Arbitrary units)
0ng/μL
10ng/μL

Figure S6. Identification of phosphatidylethanolamine (PE) species by LC-MS/MS. Related to Figure 6.

(A) Relevant section of the base peak chromatogram of control and Pla2g1b-treated larvae showing elution of PE phospholipids in negative ion mode, n=3. (B) Example spectrum form the section of the chromatogram in (A) at the approximate retention time of PE species 23.23-23.40 minutes. PEs consist of a phosphoethanolamine head group attached to a glycerol backbone, itself attached to two fatty acid moieties via phosphoether and ester bonds, respectively. As a result, PE MS spectra display traits of fatty acid-containing molecules (clusters that have inter-cluster mass shifts of 28 Da (CH_2CH_2) and intra-cluster mass shifts of 2 Da (indicative of difference in double bond number (fatty acid saturation)). The six identified and three putatively identified PEs are shown in Figure 6A, B. (C) Extracted ion chromatogram of the ion corresponding to PE 36:3 (18:1, 18:2) in negative ion mode (m/*z* 740.5226). (D) Positive-ion MS/MS was used to confirm assignment of the peak as PE, where neutral loss of 141 Da indicates phopshoethanolamine head group. Here, we show the fragmentation of the ion corresponding to PE 36:3 (18:1, 18:2) in positive ion mode (m/*z* 742.5362). (E) Negative ion fragmentation of the ion corresponding to PE 36:3 (18:1, 18:2) (m/*z* 740.5226). By identifying both fatty moieties, the individual molecular species were recognized (in this instance, 18:1 and 18:2). Both, the arrangement of the fatty acid moieties at the glycerol backbone (i.e. *sn*-1 or *sn*-2) and the position of the double bonds could not be inferred. (F) Relative abundances of unidentified lipid features extracted from PLA_2g1B-treated (10 ng/µL) and control-treated (0 ng/µL), n=3. Data is shown as normalised intensities expressed in arbitrary units. Data represented as mean ± SEM. TIC: Total ion current.

Cluster 1: Genes	*H.p.* 2º vs *H.p.* 1º Fold change	*H.p.* 1º Fold change (rel. to naïve)	*H.p.* 2º Fold change (rel. to naïve)
Chi3l3/Chi3l4	31.57018	126.558	3995.459
GSDMC	17.25631	67.532	1165.353
SPP1	12.70641	5.024	63.837
RNASE2	12.13344	373.801	4535.492
Retnlb	7.871389	66.013	519.614
Ccl8	7.796338	11.632	90.687
PLA2G4C	7.76945	49.434	384.075
Retnla	7.459337	73.334	547.023
ARG1	5.484869	24.023	131.763
TIMP1	5.245189	11.432	59.963
OLR1	5.064237	5.542	28.066
CCL24	4.347589	14.474	62.927
Irg1	3.618186	17.986	4.971
CCL7	3.5964	8.444	30.368
F7	3.310224	25.127	83.176
CPXM1	3.203643	5.161	16.534
Ccl2	2.565859	8.465	21.72
HP	2.459766	12.502	30.752
Ear2 (includes others)	2.434257	5.126	12.478
F13A1	2.262129	5.524	12.496
TREM2	2.22541	8.225	18.304
Cbr2	2.195861	10.533	23.129
SERPINA3	2.081023	8.368	17.414
C3	2.007541	5.172	10.383
Saa3	1.967481	9.779	19.24
2310042E22Rik (includes others)	1.731672	11.758	20.361
Scd2	1.318527	5.729	4.345
SELL	1.05153	8.694	9.142

Table S1. Cluster 1 gene list. Related to Figure 1.

Genes in cluster 1 as determined in Fig. 1c (Filtered on >5 fold in *H.p.* 1º (relative to naïve), $p<0.05$).

**Cluster 2: Genes**	***H.p.* 2º vs *H.p.* 1º Fold change**	***H.p.* 1º Fold change (rel. to naïve)**	***H.p.* 2º Fold change (rel. to naïve)**
1810009J06Rik/Gm2663	162.088	1	162.088
Mcpt1	74.305	1	74.305
Mcpt2	59.661	1	59.661
REG1B	45.654	1	45.654
Reg3d	33.858	1	33.858
CPA3	32.831	1	32.831
SPTA1	28.655	1	28.655
mir-675	26.276	1	26.276
FETUB	22.682	1	22.682
Rab44	19.101	1	19.101
PPY	17.489	1	17.489
SLC7A2	17.028	1	17.028
RANBP3L	16.868	1	16.868
PAH	16.531	1	16.531
CUZD1	16.173	1	16.173
ITIH4	15.567	1	15.567
PRSS3	14.71	1	14.71
SPRR2G	12.941	1	12.941
AMY2A	12.738	1	12.738
Prss2	12.642	1	12.642
PAQR9	12.445	1	12.445
Try4/Try5	12.386	1	12.386
DCDC2	12.242	1	12.242
GC	11.945	1	11.945
Tmed11	11.423	1	11.423
GGH	11.2	1	11.2
SYCN	11.142	1	11.142
CEL	11.14	1	11.14
SERPINI2	10.909	1	10.909
AMY1A (includes others)	10.779	1	10.779
CCL2	10.591	1	10.591
KLK3	10.557	1	10.557
GP2	10.119	1	10.119
CELA3A	9.918	1	9.918
PTF1A	9.807	1	9.807
SOSTDC1	9.777	1	9.777
CTRB2	9.712	1	9.712
CPA1	9.672	1	9.672
TMED6	9.625	1	9.625
CCKAR	9.609	1	9.609
Gm5409/Try10	9.585	1	9.585
CLDN10	9.084	1	9.084
AASS	8.706	1	8.706
RNASE1	8.567	1	8.567
PTGER3	8.199	1	8.199
SLC38A3	8.189	1	8.189
PNLIPRP2	7.977	1	7.977
CBS	7.932	1	7.932

RETNLB	7.896	1	7.896
Amy2b	7.83	1	7.83
ALOX15	7.823	1	7.823
CLPS	7.77	1	7.77
LOC100862462	7.684	1	7.684
Siglec5	7.652	1	7.652
GATM	7.619	1	7.619
ERP27	7.596	1	7.596
ANGPT1	7.54	1	7.54
SLC34A2	7.502	1	7.502
TNIP3	7.492	1	7.492
MUC6	7.477	1	7.477
CPB1	7.448	1	7.448
C8orf47	7.352	1	7.352
CASP9	7.349	1	7.349
SH2D6	7.318	1	7.318
PLA2G1B	7.292	1	7.292
LRRC7	7.23	1	7.23
HAPLN4	7.132	1	7.132
Igk	7.123	1	7.123
CHST2	7.106	1	7.106
NUPR1	6.785	1	6.785
RGS22	6.752	1	6.752
VTN	6.715	1	6.715
PNLIPRP1	6.651	1	6.651
AQP12A/AQP12B	6.598	1	6.598
GABRA4	6.592	1	6.592
IAPP	6.59	1	6.59
LRRN1	6.483	1	6.483
Ighg2c	6.409	1	6.409
ARHGDIG	6.349	1	6.349
Hamp/Hamp2	6.31	1	6.31
IGF1	6.273	1	6.273
TMEM108	6.273	1	6.273
TFF2	6.261	1	6.261
Wfdc18	6.256	1	6.256
Ang2 (includes others)	6.214	1	6.214
TREML2	6.161	1	6.161
LY6G6F	6.114	1	6.114
Clu	6.113	1	6.113
CTSV	6.068	1	6.068
SH2D7	6.062	1	6.062
CELA3B	6.053	1	6.053
CILP	5.948	1	5.948
ALOX5AP	5.947	1	5.947
NPHS1	5.924	1	5.924
GNMT	5.894	1	5.894
DHRS9	5.881	1	5.881
LCAT	5.863	1	5.863

FUT2	5.862	1	5.862
PRSS1	5.857	1	5.857
ALDH1L2	5.8	1	5.8
GPRC5C	5.729	1	5.729
FKBP11	5.711	1	5.711
MUC1	5.71	1	5.71
RNY1	5.698	1	5.698
HCK	5.663	1	5.663
TFPI2	5.645	1	5.645
G6PC	5.643	1	5.643
Igh-VS107	5.63	1	5.63
PDCD1LG2	5.628	1	5.628
CPA2	5.532	1	5.532
HPN	5.453	1	5.453
CLEC7A	5.45	1	5.45
GAL	5.399	1	5.399
RBPJL	5.322	1	5.322
NFIL3	5.276	1	5.276
SRGN	5.231	1	5.231
CLDN8	5.224	1	5.224
FAM221A	5.212	1	5.212
HSBP1L1	5.188	1	5.188
CELA1	5.129	1	5.129
ME1	5.12	1	5.12
CA4	5.097	1	5.097
TSPAN6	5.083	1	5.083
FN1	5.08	1	5.08
SLC38A5	5.065	1	5.065
GLIPR2	5.031	1	5.031

Table S2. Cluster 2 gene list. Related to Figure 1.

Genes in cluster 2 as determined in Fig. 1c (Filtered on >5 fold in *H.p.* 2° (relative to naïve), *p*<0.05).

**Cluster 3: Genes**	***H.p.* 2º vs *H.p.* 1º Fold change**	***H.p.* 1º Fold change (rel. to naïve)**	***H.p.* 2º Fold change (rel. to naïve)**
Gm11194	-33.158	1	-33.158
SLC13A2	-20.622	1	-20.622
DBP	-11.072	1	-11.072
Cyp2a12/Cyp2a22	-7.598	1	-7.598

Table S3. Cluster 3 gene list. Related to Figure 1.

Genes in cluster 3 as determined in Fig. 1c (Filtered on <-5 fold in *H.p.* 2º (relative to naïve), $p<0.05$).

Gene	Forward primer sequence	Reverse primer sequence
Hprt	GCCCTTGACTATAATGAGTACTTCAGG	TTCAACTTGCGCTCATCTTAGG
Pla2g1b	CTCGGGCCGTGTGGCAGTTC	TGCCGAGCCAGAGCACGAGT
Arg1	GGAAAGCCAATGAAGAGCTG	GCTTCCAACTGCCAGACTGT
Retnla	CCCTCCACTGTAACGAAGACTC	CACACCCAGTAGCAGTCATCC
Chi3l3	CATGAGCAAGACTTGCGTGAC	GGTCCAAACTTCCATCCTCCA
Retnlb	ATGGGTGTCACTGGATGTGCTT	AGCACTGGCAGTGGCAAGTA
Gob5	CATCGCCATAGACCACGACG	TTCCAGCTCTCGGGAATCAAA

Table S4. qPCR oligonucleotide primer sequences. Related to Experimental Procedures.
